# Personalized prediction of SARS-CoV-2 vaccine-induced immunity after boost: a longitudinal analysis using joint modeling

**DOI:** 10.3389/fimmu.2025.1619631

**Published:** 2025-09-30

**Authors:** Iraklis Papadopoulos, Anh Nguyet Diep, Joey Schyns, Claire Gourzones, Frédéric Minner, Germain Bonhomme, M. Paridans, Nicolas Gillain, Eddy Husson, Mutien Garigliany, Gilles Darcis, Daniel Desmecht, Michèle Guillaume, Fabrice Bureau, Anne-Françoise Donneau, Laurent Gillet

**Affiliations:** ^1^ Biostatistics and Research Method Center - Public Health Department, Liège University, Liège, Belgium; ^2^ Laboratory of Cellular and Molecular Immunology, GIGA Institute, Liège University, Liège, Belgium; ^3^ COVID-19 Platform, Liège University, Liège, Belgium; ^4^ Laboratory of Immunology-Vaccinology, FARAH, Liège University, Liège, Belgium; ^5^ Department of Pathology, FARAH, Liège University, Liège, Belgium; ^6^ From Biostatistics to Health Promotion Research Unit, Public Health Department, Liège University, Liège, Belgium; ^7^ Infectious Diseases Department, Centre Hospitalier Universitaire de Liège, Liège, Belgium

**Keywords:** joint modelling, SARS-CoV-2, immune response, breakthrough infection, prediction

## Abstract

**Introduction:**

The SARS-CoV-2 pandemic has revealed substantial inter-individual variability in immune responses, particularly following widespread primary vaccination and booster campaigns. These differences affect the durability of protective immunity and the need for additional booster doses. To optimize the management of current and future epidemics, there is a critical need for predictive tools that personalize immune monitoring and guide targeted booster strategies for vulnerable populations.

**Methods:**

In this study, we conducted a 15-month longitudinal analysis of a cohort of 1,000 individuals to identify key determinants of serological response following the first SARS-CoV-2 vaccine booster. We investigated how these factors influenced the risk of subsequent infection, and we developed statistical models to predict individual trajectories of anti-spike (S) IgG and neutralizing antibody (NAb) levels.

**Results-discussion:**

Our findings show that joint models (JMs), which integrate longitudinal antibody measurements with infection outcomes, significantly outperform traditional modeling approaches in predicting immune trajectories. This work underscores the potential of joint modeling to enable personalized immune surveillance, supporting strategies to sustain protective immunity in high-risk populations. In the future, this approach may be adapted for monitoring long-term immunity against other infectious diseases.

## Introduction

Five years after the first recorded COVID-19 occurrence (1), at the end of 2024, 777 million cases were confirmed as positive with 7.09 million deaths attributed to the disease (2). In front of this massive burden, vaccination strategies against SARS-CoV-2 played a key role in managing the pandemic. To date, 13.64 billion vaccine doses have been administered worldwide, with 67% of the total population considered to have completed the primary series of vaccination (3). In Belgium, at the end of 2024, 4.9 million COVID-19 cases have been officially reported since the beginning of the pandemic, resulting in 34.3 thousand deaths. Meanwhile, 76% of the population has received at least a complete primary vaccination scheme and 60% a first booster dose, while only 33% and 16% have received a second and a third one, respectively (4). We are now in a post-vaccination era characterized by different features: the waning protection of vaccines against COVID-19 (5–7), the evolution of the virus into new variants with higher transmissibility that cannot be fully covered by the vaccines (8–10), and vaccine hesitancy (11–13). Personalized follow-up is therefore essential to identify and prioritize individuals in the population who need booster vaccines.

Risk factors related to SARS-CoV-2 infection have been investigated extensively through numerous observational studies (14, 15). The BNT162b2 (Pfizer-BioNTech), mRNA-1273 (Moderna), ChAdOx1-S (Oxford-AstraZeneca), and Ad26.COV2.S (Johnson & Johnson) vaccines administered in European countries, including Belgium, have proven to be safe and effective against COVID-19 (16–19). However, these vaccines provide varying degrees of protection depending on other parameters (20) such as previous infection (21, 22), vaccination profiles (23), comorbidities, age (24), and gender.

Neutralizing antibody (NAb) titers and anti-Spike IgG (anti-S IgG) have previously been shown to correlate with protection against breakthrough infections (25–27) (defined as SARS-CoV-2 infection in vaccinated people) and vaccine effectiveness (28). Several studies have well-documented the fact that protection wanes over time, both after the primary and booster vaccinations, with variations associated with different risk factors (7, 8, 29–37). However, few studies have fully examined the occurrence of infection over time while considering the underlying waning of immunity based on NAb and anti-S IgG measurements. In particular, joint modeling could be a powerful tool to analyze the dynamic association between antibody levels and infection risk by simultaneously accounting for longitudinal changes and time-to-event data (38, 39). Unlike traditional models, joint models (JMs) have the potential to improve predictive accuracy by accounting for the monotone missingness of antibody data post-infection and providing individualized predictions based on real-time trajectories (38, 40). In the end, as predictions can be updated as more information becomes available, this approach could guide targeted booster strategies in real-time and on a personalized basis.

The aim of the present study was therefore to examine the evolution of anti-S IgG and Nabs following a booster dose while concurrently monitoring the occurrence of infection in order to identify post-booster risk factors for breakthrough SARS-CoV-2 infections over time and, ultimately, to develop a statistical model for prioritizing individuals for subsequent revaccination based on individualized predictions for antibody responses.

## Materials and methods

### Study design and participants

SARSSURV-ULiège (41) is a prospective, longitudinal, university population-based cohort study approved by the University Hospital of Liège Ethics Committee (reference number 2021/96 dated 26 March 2021). After providing written participation consent, students and staff members, over 18 years old, both vaccinated and unvaccinated, were enrolled and followed up from April 2021 to December 2022. Upon registration, they were required to fill in anamnestic and clinical questionnaires for medical history and treatments undertaken. At the time of enrollment, participants declared previous infection (confirmed by a serological, antigenic, a PCR test) and the respective dates of test positivity and vaccination status. During the study, weekly saliva samples were collected to perform quantitative reverse transcriptase PCR (RT-qPCR) to detect SARS-CoV-2 infection until 30 June 2022. Participants could also declare an infection to the research team via email or phone. In addition to weekly saliva samples, blood samples were collected longitudinally after an antigen exposure (vaccination or infection) to measure SARS-CoV-2 NAb and anti-S IgG concentrations. In order to identify undeclared or asymptomatic infections that may have occurred before or during the study period despite the weekly PCR screening, anti-SARS-CoV-2 nucleocapsid (anti-N) serological analyses were performed on all serum samples. Participants who presented a positive anti-N result at the first blood sample after booster (2 weeks after) were considered previously infected. Detection of infection through anti-N serology was not performed before the primary vaccination due to the fact that blood samples were only collected after the primary vaccination. Additionally, we considered the occurrence of an unreported infection when an increase of more than 40% in anti-S IgGs or NAb was observed after a recorded decrease of those concentrations at the previous blood sampling. In such cases, participants with unreported infections were followed up until the confirmed date, indicating that they were uninfected, as the exact date of infection could not be inferred. The current study involved participants who had been fully vaccinated with either two-dose (BNT162b2, mRNA-1273, and ChAdOx1-S) or one-dose (Ad26.COV2.S) vaccines, boosted by an mRNA vaccine (BNT162b2 and mRNA-1273), and had at least one blood sample taken after the booster dose.

### SARS-CoV-2 blood sampling

Serum samples were collected after each exposure to SARS-CoV-2 antigens (primary vaccination, booster, and/or infection), more specifically, during period 1 (between 15 and 45 days after the exposure) and approximately every 3 months thereafter, namely, period 2 (between 90 and 120 days after the exposure), period 3 (between 180 and 210 days after the exposure), and, for a certain number of participants who had not received a second booster or were not infected after the booster, period 4 (between 270 and 310 days after the exposure) and period 5 (between 360 and 400 days after the exposure). The planned schedule for serum collection was reset after each new exposure. We focused on serum samples after the booster dose that corresponded only to the booster exposure (visiting process in **Supplementary Figure S2**, **Supplementary Material**).

### Anti-SARS-CoV-2 antibody quantification

#### Measurement of anti-SARS-CoV-2 Spike IgG and total anti-SARS-CoV-2 nucleocapsid antibodies

Anti-S IgGs were quantified using a chemiluminescent immunoassay (LIAISON SARS-CoV-2 TrimericS anti-Spike IgG detection kit, DiaSorin, Stillwater, MN, USA). Luminescence was detected using a LIAISON XL analyzer (DiaSorin). The readout was expressed in binding antibody units/mL (BAU/mL) by applying the conversion factor recommended by the manufacturer (BAU/mL = U/mL * 2.6), and a sample was considered positive if the anti-S IgG concentration was ≥33.8 BAU/mL. Anti-N antibodies were detected using the Elecsys anti-SARS-CoV-2 electrochemiluminescence immunoassay on a Cobas e411 (Roche Diagnostics GmbH, Mannheim, Germany). This assay reports results in a dimensionless cut-off index (COI), with values<1.0 COI indicating negative and ≥1.0 COI positive. Both tests were performed according to the manufacturer’s instructions in accredited clinical laboratories (University Hospital of Liège, Belgium).

#### Neutralizing antibody quantification

Virus neutralization test (VNT) was carried out with SARS-CoV-2 strain BetaCov/Belgium/Sart-Tilman/2020/1 in 96-well plates containing Vero E6 cells (ATCC CRL-1586). Six dilutions of each heat-inactivated serum were used (1:10 to 1:320, corresponding to final testing dilutions 1:20 to 1:640). In each VNT, a positive control serum from the Belgian National Reference Centre (Sciensano, Rue Juliette Wytsman 14, Ixelles, Brussels) was used. Sera were mixed vol/vol with 100 TCID_50_/reaction of SARS-CoV-2 virus and incubated at 37 °C for 1 hour. Then, the serum plus virus mixture was transferred to the cells in suspension in six replicates. The VNT relies on cytopathic effect (CPE) observation under light microscopy at day 5 post-infection. Dilutions of serum associated with CPE were considered negative, while the absence of CPE indicated a complete neutralization of SARS-CoV-2 inoculum (positive). Virus neutralization titer was reported as the highest dilution of serum that neutralized CPE in 50% of the wells.

### Risk factors

Participant characteristics included age at booster vaccination (year), gender, smoking behavior, body mass index (BMI; kg/m^2^), blood group, and rhesus status. Clinical characteristics included information about comorbidities such diabetes, hypertension, heart insufficiency, stroke attack, asthma, pulmonary disease, antécédents cardiaques, history of heart disease (ATC)-cardiac (presence of at least one of the following: diabetes, hypertension, stroke, and heart failure), ATC-pulmonary (presence of at least one of the following: pulmonary disease and asthma), medication intake, autoimmune diseases, renal failure, and immunosuppression. The types of primary vaccination [mRNA (BNT162b2 or mRNA-1273) or adenovirus vector-based vaccines (ChAdOx1-S or Ad26.COV2.S)] and booster dose (BNT162b2 and mRNA-1273) were also collected. Weekly incidence data were extracted from the national database for COVID-19 monitoring for the province of Liège, to be explored as potential exogenous risk factors and assigned to participants’ date of blood sampling, and weekly positivity (%) of the SARSSURV cohort was obtained based on weekly saliva testing of the university population. The booster vaccination period was defined based on 31 December 2021 (before and afterward), which was the date corresponding to the start of dominance of the Omicron variant in Belgium (4).

### Outcomes

Three outcomes were investigated. Namely, we examined the decline following the booster dose of both anti-S IgG and SARS-CoV-2 NAb levels. Additionally, we focused on the time to the first indication of SARS-CoV-2 breakthrough infection after the booster dose. We identified breakthrough infections after the booster through positive saliva tests or confirmed self-declared PCR tests at least 14 days after the booster dose when immunity had developed.

### Person-time at risk

Regarding breakthrough infection, the considered follow-up period began on 15 September 2021 and continued until 22 December 2022, which was the date of the last collected SARSSURV blood sample. The date of the last booster dose was 15 July 2022. Participants were followed up from the date of their booster vaccination. We defined the end of follow-up for each participant as the date of their first documented breakthrough infection. For participants who remained uninfected, follow-up ended at the date of their last available blood sample or the date of their second booster dose.

### Statistical analysis

Participant inclusion characteristics were summarized globally and by infection status using descriptive statistics including mean and standard deviation for normally distributed quantitative variables, median and interquartile range were reported in case of non-normality, and frequencies (%) were reported for qualitative variables. Differences in inclusion variables according to the censoring status (breakthrough infection) were investigated using Student’s t-test, Mann–Whitney, and chi-square test or Fisher’s exact for the respective quantitative and qualitative variables.

Linear mixed-effects models (42) (LME) under the maximum likelihood method were applied to assess the evolution of NAb and anti-S IgG over time after the booster dose and to compare the measurements between different participants’ characteristics. NAb and anti-S IgG were modeled on a natural log scale. Group differences and quantitative variables for a one-unit increase were expressed as geometric mean ratio (GMR) with 95% confidence interval (CI) on the original scale of anti-S IgG and NAb. To account for the variability unexplained by the fixed effects, random effects were used (43). More specifically, random intercept and random slope were considered to account for individual variability in first outcome measurements and in change over time, respectively. After comparison with different degrees of freedom splines, natural splines with three degrees of freedom were retained to be applied to the time effect in every model. The normality of distribution of residuals and random effects of the final multivariable models was graphically investigated, and the absolute difference between the predicted and observed values of the longitudinal measurements was used to assess the overall performance of the LME models. The half-life (t_1/2_) value associated with NAb and anti-S IgG waning, based on univariable LME models adjusted only for time, was calculated using the formula $t_{1/2}=\frac{\text{ln}\;\left(0.5\right)}{\beta_{1}}$ (where β_1_ is the regression coefficient of the time effect) completed with the delta method for the corresponding 95% CI, and it represents the time until a 50% reduction of antibodies from the mean baseline levels.

Hazard ratios (HRs) with corresponding 95% CIs were reported for potential risk factors for time to breakthrough infection using Cox proportional hazards (Cox-PH) models. The best multivariate model was chosen to be the most parsimonious one based on the backward elimination method. The proportionality of the adjusted variables was tested using the Schoenfeld residuals and log–log plots of survival. The Kaplan–Meier plots were depicted to provide a visual representation of the significant covariates. In both longitudinal and survival models, the time origin starts at the date of the booster.

To simultaneously investigate the association between repeated outcome measurements and the risk of a breakthrough infection, two JMs for longitudinal and time to event data were applied (38), one for each longitudinal outcome. To obtain insights into the association between longitudinal measurements and the risk of breakthrough infection, the two JMs combined the multivariate Cox-PH model with each of two LME models for NAb and anti-S IgG described previously. Individual longitudinal predictions for NAb and anti-S IgG were derived from these JMs, and two longitudinal predictions with 95% CI for two randomly selected participants are presented. JMs were performed under the Bayesian framework using Markov Chain Monte Carlo (MCMC) methods with Gibbs sampling, and results were summarized from five chains, each consisting of 20,000 MCMC iterations, after discarding 2,000 burn-in samples per chain. The survival sub-model was parameterized with the current value association, such that the hazard of breakthrough infection at time *t* depended on the predicted antibody level at time *t* from the longitudinal sub-model. We chose the current value because the antibody level at the time of exposure is the most clinically relevant determinant of infection risk. No use of other functions of antibodies was made.

Model selection for the LME model, Cox-PH model, and JM was assessed using Akaike information criterion (AIC), log-likelihood, and deviance information criterion (DIC). The overall performance of the longitudinal sub-models was assessed by calculating the mean absolute error (MAE) and the root mean square error (RMSE) between the predicted and observed log(IgG) and log(NAb) measurements. Individualized predictions were obtained from the joint model using empirical Bayes estimates of the subject-specific random effects, conditional on each participant’s antibody history up to the prediction time. These, therefore, reflect personalized trajectories rather than marginal (population-average) predictions. This predictive performance measure described above was obtained using a cross‐validation procedure (internal validation). The data set was split into 10 subsets (10-fold cross-validation), of which nine were used to fit the model and one was used to obtain predictions (**Supplementary Figure S7**). This process was repeated 10 times, with each subset being used as the testing set once. The same procedure was followed for a simple longitudinal model, and the differences in mean absolute errors of both joint models and longitudinal models are presented. The incorporation of the time-to-event sub-model allows us to reduce bias arising from informative dropout since antibody measurements are jointly modeled with the risk of infection. However, this correction depends on the joint model being correctly specified and may not fully eliminate bias under more complex missingness mechanisms (44).

Statistical significance was considered at p-value< 0.05 in all statistical models and tests. All models ran on the complete case data, and missing values are reported for each variable. Data management, visualization, and modeling were performed using the R software (version 4.3.1) (45). R packages “survival” and “lme” were considered for survival and longitudinal models, respectively. JMs were performed using the “JMBayes2” (46) R package. Diagnostics for all models can be found in the Supplementary Material (**Supplementary Figures S8–S13**).

## Results

### Study population

A total of 966 fully vaccinated and boosted participants were included, with a median age of 39.1 (26.0–49.9) years, and among these, 580 (60.0%) were female. A total of 5,963 blood samples were collected during the SARSSURV study, and 1,845 of these blood samples were kept as they were taken after the booster vaccine dose (**Supplementary Figure S1**). The median number of serum samplings for the study follow-up was 2 [interquartile range (IQR), 1–3]. In total, 31,542 SARS-CoV-2 detection salivary tests were performed by these participants during the SARSSURV study, among which 11,914 were performed during their follow-up period after the booster vaccine dose. The median number of salivary tests per participant for the study follow-up was 13 (IQR, 6–20). Baseline characteristics for demographics, comorbidities, antigen exposures, and vaccination information are summarized in **Table 1**.

**Table 1:** Participants’ characteristics, globally and according to breakthrough infection status.

Variables	Categories	Missing N (%)	Total (n = 966)	Breakthrough infection		P-value
				No (n = 619)	Yes (n = 347)	
Participant’s characteristics						
Age at vaccination (years)	Mean (SD)	0 (0.0%)	38.8 (13.4)	37.4 (13.7)	41.4 (12.4)	<0.001^1^
	Median (Q1, Q3)		39.1 (26.0, 49.9)	34.5 (24.5, 49.0)	43.2 (29.6, 51.4)	
Gender	Female (%)	0 (0.0%)	580 (60.0%)	365 (59.0%)	215 (62.0%)	0.362^3^
	Male (%)		386 (40.0%)	254 (41.0%)	132 (38.0%)	
Smoking	No	0 (0.0%)	892 (92.3%)	566 (91.4%)	326 (93.9%)	0.159^3^
	Yes		74 (7.7%)	53 (8.6%)	21 (6.1%)	
BMI (weight/height^2^)	Mean (SD)	0 (0.0%)	23.68 (2.5)	23.9 (2.5)	23.2 (2.3)	<0.001^1^
	Median (Q1, Q3)		23.5 (21.9, 25.2)	23.6 (22.0, 25.2)	22.8 (21.4, 24.8)	
Blood group	O	142 (14.7%)	350 (42.5%)	225 (43.1%)	125 (41.4%)	0.249^3^
	A		380 (46.1%)	230 (44.1%)	150 (49.7%)	
	B		62 (7.5%)	45 (8.6%)	17 (5.6%)	
	AB		32 (3.9%)	22 (4.2%)	10 (3.3%)	
Rhesus	Positive (+)	170 (17.6%)	656 (82.4%)	414 (82.1%)	242 (82.9%)	0.793^3^
	Negative (−)		140 (17.6%)	90 (17.9%)	50 (17.1%)	
Antigen exposure and vaccination						
Primary vaccine	Ad26.COV2.S (%)	0 (0.0%)	28 (2.9%)	13 (2.1%)	15 (4.3%)	<0.001^3^
	BNT162b2 (%)		709 (73.4%)	469 (75.8%)	240 (69.2%)	
	ChAdOx1-S (%)		150 (15.5%)	76 (12.3%)	74 (21.3%)	
	mRNA-1273 (%)		79 (8.2%)	61 (9.9%)	18 (5.2%)	
Primary vaccine type	Adenovirus vectored (%)	0 (0.0%)	178 (18.4%)	89 (14.4%)	89 (25.6%)	<0.001^3^
	mRNA (%)		788 (81.6%)	530 (85.6%)	258 (74.4%)	
Booster	BNT162b2	0 (0.0%)	774 (80.1%)	485 (78.4%)	289 (83.3%)	0.06^3^
	mRNA-1273		192 (19.9%)	134 (21.6%)	58 (16.7%)	
Booster vaccination season	Before 31 Dec 2021	0 (0.0%)	594 (61.5%)	341 (55.1%)	253 (72.9%)	0.193^3^
	After 31 Dec 2021		372 (38.5%)	278 (44.9%)	94 (27.1%)	
Infection before booster	No (%)	0 (0.0%)	654 (67.7%)	383 (61.9%)	271 (78.1%)	<0.001^3^
	Yes (%)		312 (32.3%)	236 (38.1%)	76 (21.9%)	
Infection before primary vaccination	No (%)	0 (0.0%)	772 (79.9%)	484 (78.2%)	288 (83.0%)	0.07^3^
	Yes (%)		194 (20.1%)	135 (21.8%)	59 (17.0%)	
Infection between primary and booster vaccinations	No (%)	0 (0.0%)	871 (90.2%)	544 (87.9%)	327 (94.2%)	<0.001^3^
	Yes (%)		95 (9.8%)	75 (12.1%)	20 (5.8%)	
Delay between primary-vaccination doses (days)	Mean (SD)	0 (0.0%)	184 (33.6)	186 (36.3)	180 (27.9)	<0.001^2^
	Median (Q1, Q3)		182 (163, 196)	182 (164, 197)	179 (161, 193)	
Time since booster from last blood sample before	Mean (SD)		48.5 (29.8)	47.9 (29.6)	49.6 (30.1)	0.560^1^
	Median (Q1, Q3)		51.0 (23.0, 71.0)	51.0 (22.0, 71.0)	54.0 (24.5, 71.0)	
Neutralizing antibody levels before the booster dose	[0, 160)	57 (5.9%)	644 (70.8%)	370 (63.9%)	274 (82.8%)	<0.001^1^
	[160, 320)		93 (10.2%)	70 (12.1%)	23 (6.9%)	
	[320, 1,280)		128 (14.1%)	98 (16.9%)	30 (9.1%)	
	[1,280, +)		45 (5.8%)	41 (7.1%)	4 (1.2%)	
Anti-S IgG (BAU/mL) levels before the booster dose	[0, 1,500)	58 (6.0%)	699 (76.9%)	409 (70.8%)	290 (87.6%)	<0.001^1^
	[1,500, 3,370)		126 (13.9%)	99 (17.1%)	27 (8.2%)	
	[3,370, 6,532)		46 (5.1%)	36 (6.2%)	10 (3.0%)	
	[6,532, +)		38 (4.2%)	34 (5.9%)	4 (1.2%)	
Baseline neutralizing antibody levels (2 weeks after booster date)	[0, 160)	0 (0.0%)	48 (5.0%)	26 (4.2%)	22 (6.3%)	<0.001
	[160, 320)		113 (11.7%)	55 (8.9%)	58 (16.7%)	
	[320, 1,280)		404 (41.8%)	232 (37.5%)	172 (49.6%)	
	[1,280, +)		401 (41.5%)	306 (49.4%)	95 (27.4%)	
Baseline anti-S IgG (BAU/mL) levels (2 weeks after booster date)	[0, 1,500)	0 (0.0%)	65 (6.7%)	40 (6.5%)	25 (7.2%)	<0.001
	[1,500, 3,370)		205 (21.2%)	114 (18.4%)	91 (26.2%)	
	[3,370, 6,532)		309 (32.0%)	172 (27.8%)	137 (39.5%)	
	[6,532, +)		387 (40.1%)	293 (47.3%)	94 (27.1%)	
Comorbidities						
Diabetes	No (%)	26 (2.7%)	926 (98.5%)	594 (98.7%)	332 (98.2%)	0.585^4^
	Yes (%)		14 (1.5%)	8 (1.3%)	6 (1.8%)	
Hypertension	No (%)	29 (3.0%)	855 (91.2%)	549 (92.1%)	306 (89.7%)	0.215^3^
	Yes (%)		82 (8.8%)	47 (7.9%)	35 (10.3%)	
Heart failure	No (%)	26 (2.7%)	930 (98.9%)	595 (98.8%)	335 (99.1%)	0.693^3^
	Yes (%)		10 (1.1%)	7 (1.2%)	3 (0.9%)	
Stroke history	No (%)	2 (0.2%)	958 (99.4%)	614 (99.4%)	344 (99.4%)	0.895^4^
	Yes (%)		6 (0.6%)	4 (0.6%)	2 (0.6%)	
Asthma	No (%)	18 (1.9%)	862 (90.9%)	549 (90.0%)	313 (92.6%)	0.181^3^
	Yes (%)		86 (9.1%)	61 (10.0%)	25 (7.4%)	
Pulmonary disease	No (%)	7 (0.7%)	945 (98.5%)	604 (98.4%)	341 (98.8%)	0.780^3^
	Yes (%)		14 (1.5%)	10 (1.6%)	4 (1.2%)	
Comorbidities	None (%)	0 (0.0%)	726 (75.2%)	469 (75.8%)	257 (74.1%)	0.370^3^
	One (%)		126 (13.0%)	74 (12.0%)	52 (15.0%)	
	At least two (%)		114 (11.8%)	76 (12.3%)	38 (11.0%)	
ATC-cardiac	No (%)	0 (0.0%)	811 (84.0%)	523 (84.5%)	288 (83.0%)	0.543^3^
	Yes (%)		155 (16.0%)	96 (15.5%)	59 (17.0%)	
ATC-pulmonary	No (%)	0 (0.0%)	855 (88.5%)	543 (87.7%)	312 (89.9%)	0.305^4^
	Yes (%)		111 (11.5%)	76 (12.3%)	35 (10.1%)	
Medicines	No (%)	0 (0.0%)	539 (55.8%)	362 (58.5%)	177 (51.0%)	0.024^3^
	Yes (%)		427 (44.2%)	257 (41.5%)	170 (49.0%)	
Anticoagulant	No (%)	539 (55.8%)	415 (96.7%)	246 (95.7%)	167 (98.2%)	0.176^4^
	Yes (%)		14 (3.3%)	11 (4.3%)	3 (1.8%)	
Autoimmune disease	No (%)	16 (1.7%)	889 (93.6%)	572 (94.4%)	317 (92.2%)	0.176^3^
	Yes (%)		61 (6.4%)	34 (5.6%)	27 (7.8%)	
Renal failure	No (%)	10 (1.0%)	951 (99.5%)	609 (99.5%)	342 (99.4%)	0.851^3^
	Yes (%)		5 (0.5%)	3 (0.5%)	2 (0.6%)	
Immunosuppressive	No (%)	539 (55.8%)	415 (97.2%)	248 (96.5%)	167 (98.2%)	0.377^4^
	Yes (%)		12 (2.8%)	9 (3.5%)	3 (1.8%)	

^1^Mann–Whitney test.

^2^t-Test.

^3^Chi-square test.

^4^Fisher’s exact test.

At the time of the booster, 709 participants (73.4%) were previously primary-vaccinated by BNT162b2, while 79 (8.2%) were vaccinated by mRNA-1273, 150 (15.5%) by ChAdOx1-S, and 28 (2.9%) by Ad26.COV2.S. In total, 788 (81.6%) participants were primary-vaccinated by an mRNA-based vaccine. Most of the participants were boosted with a BNT162b2 (80.1%) booster dose. After 31 December 2021, which was the starting point of the Omicron variant dominance in Belgium, a total of 372 (38.5%) participants received the booster dose, literally during the Omicron wave outbreak. At the time of booster vaccination, 312 (32.3%) participants were previously SARS-CoV-2 infected. During the follow-up of the boosted participants, 347 (35.9% of the participants) SARS-CoV-2 infections were recorded. The median follow-up period was 172 (IQR, 85–281) days, while the median time to breakthrough infection was 104 (IQR, 53–165) days.

### Waning of the anti-SARS-CoV-2 immune response

**Figure 1:**
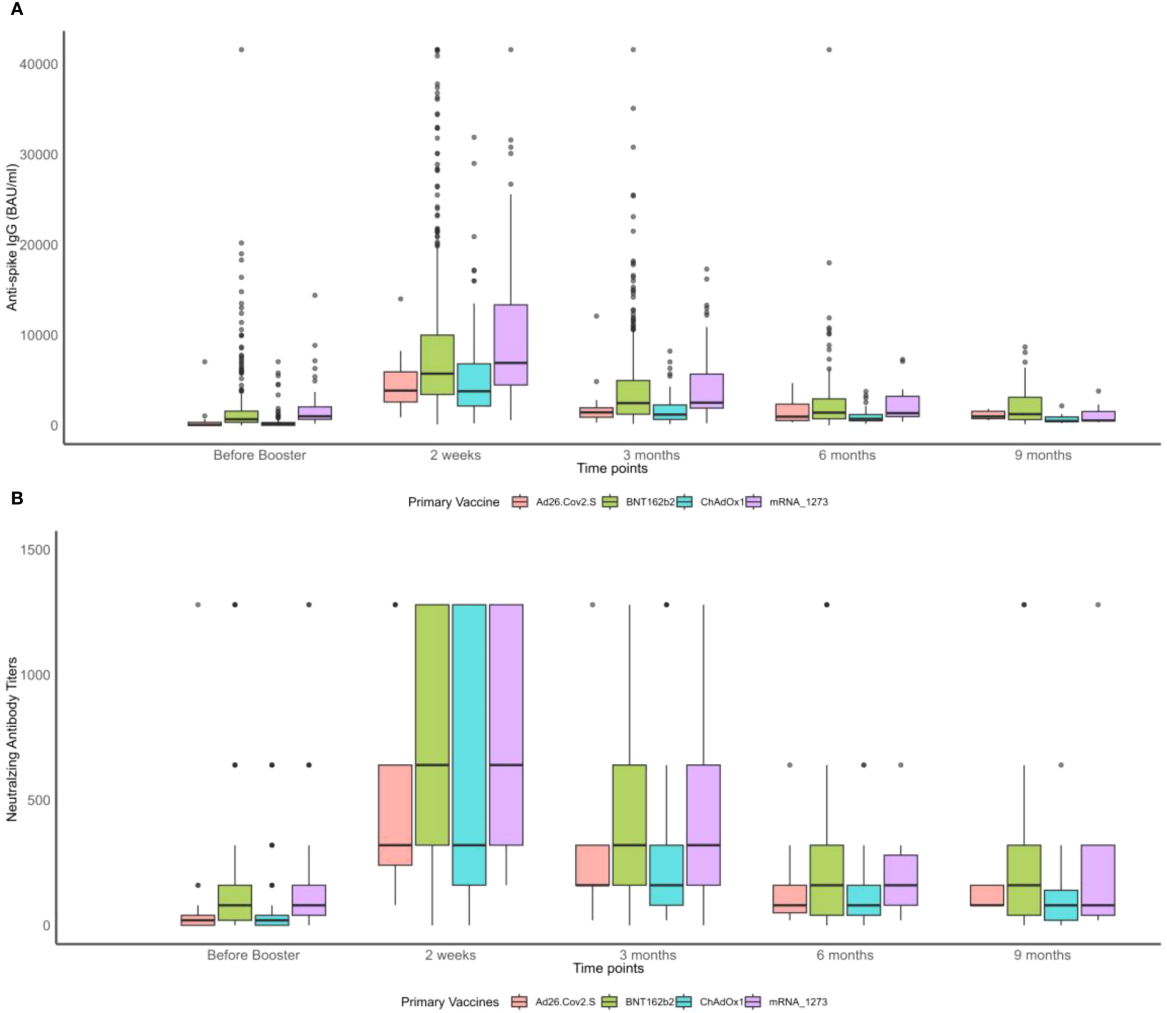
Boxplots of neutralizing antibody (NAb) titers and anti-S IgG before and after the booster vaccination by primary vaccine type over time. **(A)** Anti-spike IgG measurements. **(B)** Neutralizing antibody titers.

**Figure 2:**
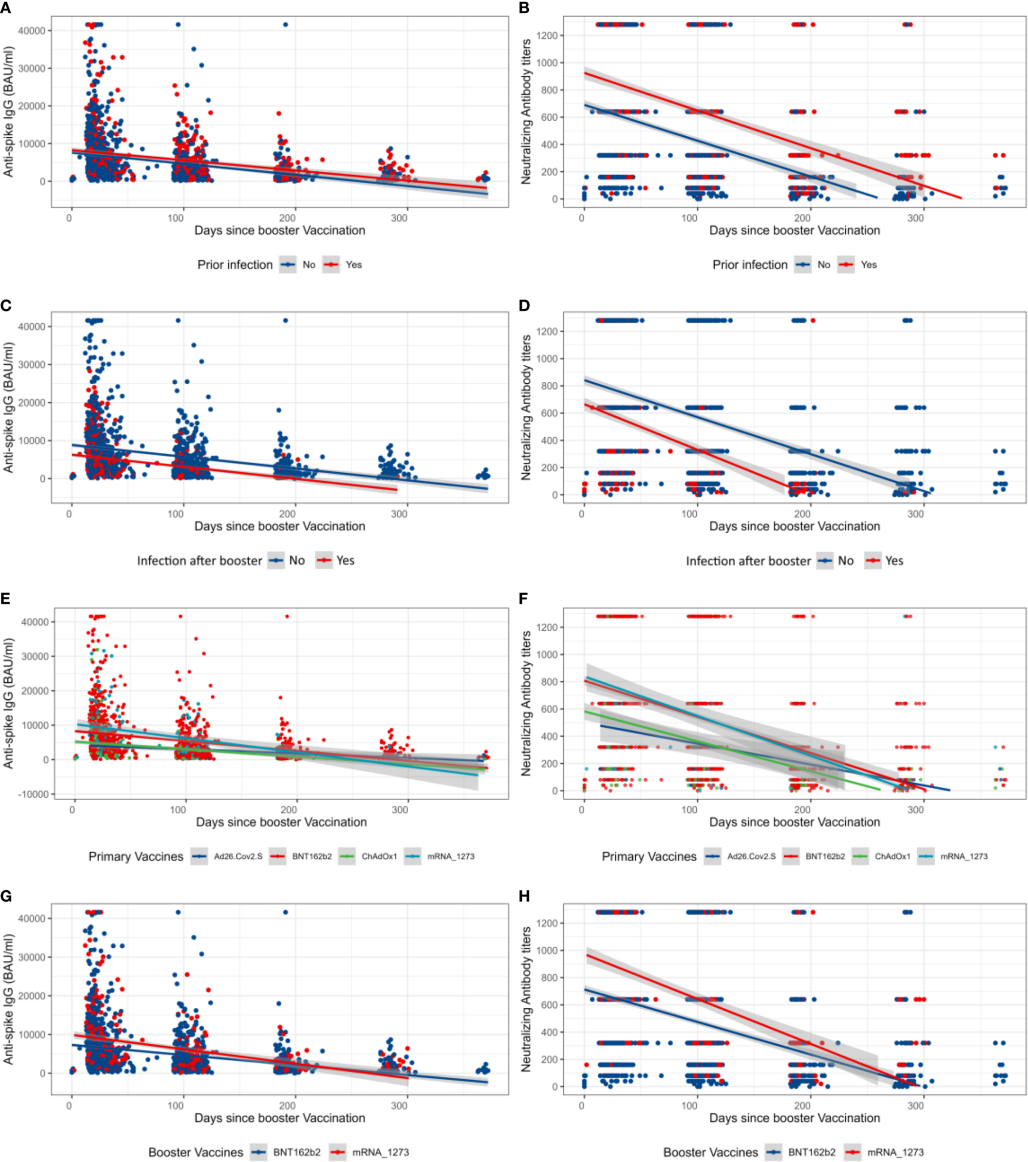
Overall longitudinal changes after the booster vaccination of neutralizing antibody titers and anti-Spike IgG. **(A, B)** Anti-S IgG and neutralizing antibody (NAb) values after the booster dose prior to booster infection status. **(C, D)** Anti-S IgG and NAb values after the booster dose of participants who were infected or not after the booster dose. **(E, F)** Anti-S IgG and NAb values after the booster dose by primary vaccination vaccine types. **(G, H)** Anti-S IgG and NAb values after the booster dose by booster type. The shaded areas represent the 95% confidence interval.

As shown in **Figure 1**, NAb and anti-S IgG waned over time, as already observed in the whole cohort (47). Participants primarily vaccinated with mRNA vaccines maintained significantly higher levels of NAb and anti-S IgG just before the booster (**Figure 1**). Those who reported an infection prior to the booster dose seemed to have developed higher levels of NAb and anti-S IgG compared to those who did not (**Figures 2A, B**). Interestingly, most of the participants infected after the booster dose presented lower NAb and anti-S IgG levels before the booster compared to the non-infected (**Figures 2C, D**). Primary-vaccinated participants with mRNA vaccine types developed higher levels of antibody compared to participants with the adenovirus-vectored vaccine (**Figures 2E, F**). Finally, boosting with mRNA-1273 induced a higher response than with BNT162b2 (**Figures 2G, H**). Detailed waning through time by different groups, using a Locally Estimated Scatterplot Smoothing (LOESS) smoother, can be found in Supplementary Material (**Supplementary Figures S3,S4**). Graphical representation for other variables can be found in **Supplementary Figure S5**.

**Figure 3:**
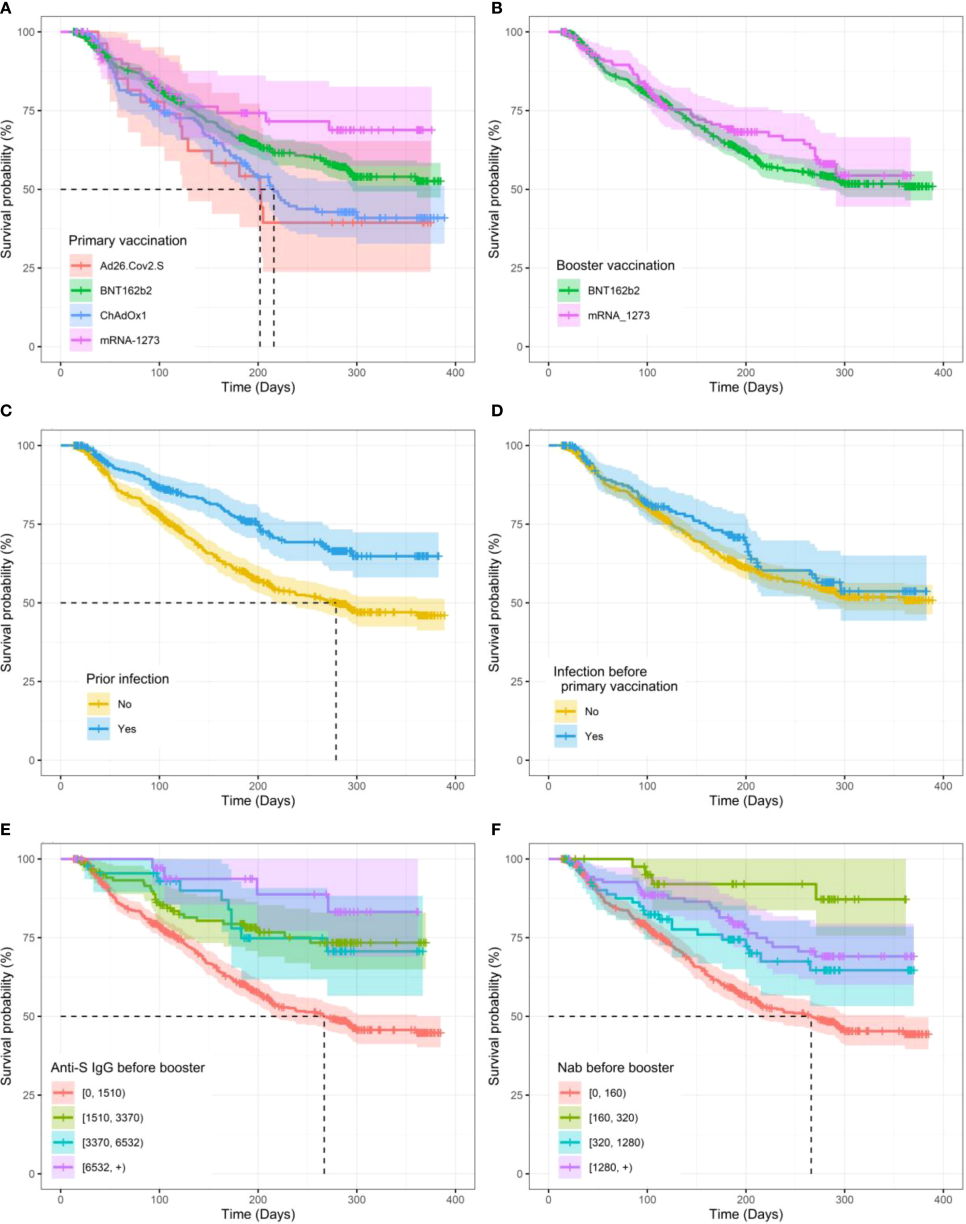
Kaplan–Meier curves for time to SARS-CoV-2 breakthrough infection after the booster vaccination. **(A)** Between primary vaccination vaccine types. **(B)** Between booster vaccination types. **(C)** Prior to infection. **(D)** Prior to infection before primary vaccination. **(E)** By levels of anti-S IgG before the booster. **(F)** By neutralizing antibody (NAb) levels before the booster. The shaded lanes represent 95% confidence intervals around the survival curve.

We then used univariate LME analyses to estimate the outcome of different variables on the levels of both NAb and anti-S IgG after the boost (**Table 2**). According to the LME models, the days when NAb and anti-S IgG levels reached 50% reduction from the peak levels were days 70 (95% CI, 67–74) and 72 (95% CI, 68–76), respectively.

**Table 2:** Univariable beta coefficient (95% CI) estimates of linear mixed-effect models for NAb and anti-S IgG after booster vaccination.

Variables	Category	Reference	Outcome: neutralizing antibodies		Outcome: anti-S IgG	
			Univariate est. (95% CI)	P-value	Univariate^1^ Est. (95% CI)	P-value
Participants’ characteristics						
Time (days)			0.99 (0.989, 0.991)	**<0.01**	**0.99 (0.989, 0.991)**	**<0.01**
Age at booster vaccination (years)			0.980 (0.976, 0.984)	**<0.01**	0.986 (0.982, 0.990)	**<0.01**
Gender	Male	Female	0.998 (0.886, 1.124)	0.973	1.032 (0.916, 1.161)	0.603
Smoking	Yes	No	0.836 (0.671, 1.041)	0.112	0.878 (0.706, 1.094)	0.244
BMI (kg/m^2^)			1.067 (1.042, 1.091)	**<0.01**	1.065 (1.041, 1.089)	**<0.01**
Blood group	O	A	0.917 (0.801, 1.050)	0.211	0.976 (0.853, 1.116)	0.730
	B		1.198 (0.934, 1.538)	0.157	1.139 (0.890, 1.460)	0.299
	AB		0.837 (0.599, 1.171)	0.302	1.025 (0.735, 1.429)	0.883
Rhesus	Positive	Negative	0.996 (0.923, 1.073)	0.907	0.99 (0.927, 1.077)	0.994
Antigen exposure and vaccination						
Infection prior to booster	**Yes**	**No**	1.411 (1.076, 1.85)	**<0.01**	1.134 (1.001, 1.284)	**0.048**
Infection prior to primary vaccination	**Yes**	**No**	1.303 (1.114, 1.525)	**<0.01**	0.808 (0.691, 0.944)	**<0.01**
Infection between primary and booster vaccinations	**Yes**	**No**	2.052 (1.683, 2.499)	**<0.01**	2.129 (1.748, 2.590)	**<0.01**
Booster vaccination Period	**After 31 Dec 2021**	**Before 31 Dec 2021**	1.782 (1.588, 1.999)	**<0.01**	1.459 (1.296, 1.641)	**<0.01**
Vaccine type	**mRNA**	**Adenovirus vectored**	1.537 (1.325, 1.782)	**<0.01**	1.629 (1.406, 1.885)	**<0.01**
Vaccine	Ad26.COV2.S	ChAdOx1-S	0.973 (0.674, 1.404)	0.884	1.016 (0.706, 1.460)	0.932
	**BNT162b2**		1.51 (1.293, 1.780)	**<0.01**	1.593 (1.36, 1.868)	**<0.01**
	mRNA-**1273**		1.647 (1.285, 2.110)	**<0.01**	2.011 (1.57, 2.572)	**<0.01**
Booster vaccine	mRNA-**1273**	**BNT162b2**	1.612 (1.397, 1.860)	**<0.01**	1.414 (1.225, 1.633)	**<0.01**
Delay between primary and booster vaccinations (days)			1.003 (1.002, 1.005)	**<0.01**	1.004 (1.002, 1.006)	**<0.01**
Liège Province weekly incidence (per 1,000)			1 (0.995, 1.005)	0.992	1.003 (0.99, 1.006)	0.157
Weekly SARSSURV positivity			1.007 (0.991, 1.024)	0.362	0.988 (0.976, 1)	0.053
Comorbidities						
Diabetes	Yes	No	0.985 (0.604, 1.603)	0.950	1.397 (0.861, 2.268)	0.176
Hypertension	Yes	No	0.835 (0.677, 1.029)	0.092	1.014 (0.822, 1.249)	0.897
Heart failure	Yes	No	1.050 (0.582, 1.892)	0.872	1.076 (0.59, 1.942)	0.806
Renal failure	Yes	No	1.052 (0.468, 2.365)	0.902	1.73 (0.77, 3.89)	0.184
Stroke history	Yes	No	0.973 (0.470, 2.015)	0.943	0.875 (0.422, 1.814)	0.721
Asthma	Yes	No	1.094 (0.891, 1.344)	0.391	1.153 (0.938, 1.417)	0.173
Pulmonary disease	**Yes**	**No**	1.462 (0.896, 2.384)	0.128	1.909 (1.172, 3.111)	**0.009**
Medication	Yes	No	0.853 (0.758, 0.959)	**0.0084**	0.934 (0.83, 1.050)	0.257
Anticoagulant	Yes	No	1.153 (0.696, 1.907)	0.580	1.543 (0.923, 2.580)	0.099
Immunosuppressive	**Yes**	**No**	0.489 (0.282, 0.846)	**0.011**	0.472 (0.27, 0.826)	**0.009**
Autoimmune disease	**Yes**	**No**	0.526 (0.414, 0.667)	**<0.01**	0.529 (0.418, 0.670)	**<0.01**
ATC-cardiac	Yes	No	0.93 (0.80, 1.10)	0.436	1.129 (0.964, 1.323)	0.131
ATC-pulmonary	Yes	No	1.118 (0.931, 1.343)	0.230	1.199 (1, 1.439)	0.051
Comorbidities	One	None	0.899 (0.755, 1.071)	0.237	1.001 (0.841, 1.191)	0.993
	At least two		1.002 (0.835, 1.203)	0.979	1.272 (1.061, 1.526)	**0.009**

BMI, body mass index; CI, confidence interval.

^1^All univariate estimates represent geometric mean ratio (GMR) and are minimally adjusted for time (days).

Bold values highlight the significant results with p-value<0.05.

First, the analyses highlighted that older ages were associated with lower development of NAb and anti-S IgG levels. In contrast, higher BMI was associated with higher levels. For every one-unit increase in the levels of BMI, the values of NAb and anti-S IgG were expected to increase by 6.7% (GMR, 95% CI, 1.067, [1.042 to 1.091], p< 0.01) and 6.5% (GMR, 95% CI, 1.065, [1.041 to 1.089], p< 0.01), respectively. No difference was observed in the NAb and anti-S IgG responses between gender, smoking, blood, and rhesus groups as presented in **Table 2**.

Second, regarding previous antigen exposure and vaccination, LME analyses revealed that prior infection was a major determinant of the levels of both NAb and anti-S IgG. Infections before booster dose were found to be related with the development of higher levels of NAb and anti-S IgG after the booster, with 41.1% (GMR, 95% CI, 1.411, [1.076 to 1.850], p< 0.01) and 13.4% (GMR, 95% CI, 1.134, [1.001 to 1.284], p< 0.01) higher values, respectively, compared to those never previously infected. This was particularly true if infection occurred after the primary vaccination. In contrast, infection before primary vaccination even led to lower anti-S IgG after the booster. The type of primary vaccine appeared also as an important factor, with mRNA vaccines in general inducing higher levels of both NAb and anti-S IgG after the boost. Interestingly, mRNA-1273 boosters (*vs*. BNT162b2) and increased delay (days) between the primary and booster vaccinations were associated with higher levels of NAb and anti-S IgG development (**Table 2**), while people who were boosted during the Omicron dominance (after 31 December 2021) maintained significantly higher values during the wave.

Finally, analysis of the influence of different comorbidities showed that immunosuppression and reported autoimmune disease were associated with lower levels of NAb and anti-S IgG development. Similarly, participants who received medication displayed 14.7% lower levels of NAb (GMR, 95% CI, 0.853, [0.758 to 0.959], p< 0.01). As observed in our previous study (48), patients suffering from pulmonary disease tended to have higher NAb and anti-S IgG levels after boost (**Table 2**).

### Risk of SARS-CoV-2 breakthrough infection

We then applied survival Cox-PH models to estimate the effect of different variables on the time to infection after the boost. **Table 3** summarizes the HRs with the corresponding 95% CI for participants’ characteristics and vaccination status, and the Kaplan–Meier curves for some significant and non-significant variables are presented in **Figure 3** and **Supplementary Figure S6**, respectively. First, regarding participants’ characteristics, results highlighted that for every one-unit increase in BMI, the risk of infection decreases by 5% (HR, 95% CI, 0.95 [0.90, 0.99], p = 0.023) (**Table 3**). No other significant association was found for other participant characteristics or comorbidities. Second, regarding antigen exposure and vaccination, it showed that participants with a prior infection (*vs*. uninfected) faced a 44.4% lower risk for a breakthrough infection (HR, 95% CI, 0.556, [0.431 to 0.718], p< 0.01). Similar to what was observed for the antibody levels, only infection between the primary and booster vaccinations was a significant protection factor with a 54.4% lower risk for a breakthrough infection (HR, 95% CI, 0.456, [0.280 to 0.742], p< 0.01). In contrast, we did not detect a significant effect for infections before primary vaccination. Importantly, participants who reported NAb titers lower than 160 and anti-S IgG lower than 1,510 BAU/mL just before the booster vaccination were at a higher risk for a breakthrough infection compared to participants with higher immunity levels. More specifically, participants with NAb over 1,280 just before the booster vaccination experienced an 81.7% lower risk of infection compared to those who reported NAb lower than 160 (HR, 95% CI, 0.183, [0.068 to 0.490], p< 0.01). Similarly, for anti-S IgG levels before the booster, participants who reported levels higher than 6,532 showed a 78.7% lower risk of infection compared to those who reported anti-S IgG values lower than 1,500 (HR, 95% CI, 0.213, [0.079 to 0.572], p< 0.01). For measures of NAb and anti-S IgG levels 14 days after booster vaccination, the risk of breakthrough infection was similarly lower for those who displayed NAb over 1,280 and anti-S IgG over 6,532 with 47.7% (HR, 95% CI, 0.523, [0.329 to 0.832], p< 0.01) and 42.5% (HR, 95% CI, 0.575, [0.370 to 0.894], p< 0.01) lower risk compared to people displaying NAb lower than 160 or anti-S IgG values lower than 1,500, respectively (**Table 3**). Similar to what was observed for antibody levels, the type of vaccine used and the time of the boost were important. Thus, primary vaccination with mRNA vaccines has been shown to be more protective compared to viral vector vaccines. Specifically, mRNA-1273- and BNT162b2-vaccinated participants had 49.3% (HR, 95% CI, 0.507, [0.303 to 0.929], p< 0.01) and 28.4% (HR, 95% CI, 0.716, [0.552 to 0.929], p = 0.012) lower risks, respectively, compared to ChAdOx1-S-vaccinated participants. No significant difference was found between participants primarily vaccinated with ChAdOx1-S and Ad26.COV2.S vaccines. Each 1-day increase in the time between the primary and booster vaccinations was associated with a 0.6% reduction in the risk of infection (HR, 95% CI, 0.994, [0.991 to 0.998], p< 0.01). Finally, a booster dose administered during the Omicron variant dominance in Belgium (*vs*. before) reduced the risk of breakthrough infection. Multivariate estimates for both NAb and anti-S IgG models and for the two Cox-PH models, adjusted for baseline NAb and anti-S IgG, respectively, can be found in **Table 4**.

**Table 3:** Univariable HR (95% CI) estimates of survival Cox-PH models for time to infection after the booster dose.

Variables	Category	Reference	Univariate^1^ HR (95% CI)	P-value
Participants’ characteristics				
Age at booster vaccination (years)			1.006 (0.998, 1.014)	0.145
Gender	Male	Female	0.93 (0.749, 1.155)	0.511
Smoking	Yes	No	0.736 (0.473, 1.144)	0.173
BMI (kg/m^2^)			**0.95 (0.90, 0.99)**	**0.023**
Blood group	O	A	0.864 (0.68, 1.094)	0.222
	B		0.618 (0.374, 1.02)	0.059
	AB		0.888 (0.468, 1.684)	0.715
Rhesus	Positive	Negative	1.018 (0.751, 1.381)	0.908
Antigen exposure and vaccination				
Infection prior to booster	**Yes**	**No**	**0.556 (0.431, 0.718)**	**<0.01**
Infection prior to primary vaccination	Yes	No	0.864 (0.653, 1.143)	0.306
Infection between primary and booster vaccinations	**Yes**	**No**	**0.456 (0.28, 0.742)**	**<0.01**
Neutralizing antibody levels before the booster dose	**[160, 320)**	**[0, 160)**	**0.630 (0.415, 0.956)**	**0.029**
	**[320, 1,280)**		**0.470 (0.322, 0.685)**	**<0.01**
	**[1,280, +)**		**0.183 (0.068, 0.490)**	**<0.01**
Anti-S IgG (BAU/mL) levels before the booster dose	**[1,500, 3,370)**	**[0, 1,500)**	**0.471 (0.320, 0.695)**	**<0.01**
	**[3,370, 6,532)**		**0.451 (0.240, 0.848)**	**<0.01**
	**[6,532, +)**		**0.213 (0.079, 0.572)**	**<0.01**
Baseline neutralizing antibody levels (2 weeks after booster date)	[160, 320)	[0, 160)	1.431 (0.876, 2.333)	0.152
	[320, 1,280)		1.109 (0.711, 1.728)	0.647
	**[1,280, +)**		**0.523 (0.329, 0.832)**	**0.006**
Baseline anti-S IgG (BAU/mL) levels (2 weeks after booster date)	[1,500, 3,370)	[0, 1,500)	1.226 (0.787, 1.909)	0.366
	[3,370, 6,532)		1.225 (0.800, 1.877)	0.349
	**[6,532, +)**		**0.575 (0.370, 0.894)**	**0.014**
Booster vaccination period	**After 31 Dec 2021**	**Before 31 Dec 2021**	**0.643 (0.507, 0.815)**	**<0.01**
Vaccine type	**mRNA**	**Adenovirus vectored**	**0.766 (0.602, 0.975)**	**0.030**
Vaccine	**Ad26.COV2.S**	**ChAdOx1-S**	**1.078 (0.619, 1.879)**	**0.789**
	**BNT162b2**		**0.716 (0.552, 0.929)**	**0.012**
	**mRNA-1273**		**0.507 (0.303, 0.849)**	**0.0097**
Booster vaccine	mRNA-1273	BNT162b2	0.88 (0.664, 1.167)	0.375
Delay between primary and booster vaccinations (days)			**0.994 (0.991, 0.998)**	**<0.01**
Comorbidities				
Diabetes	Yes	No	1.227 (0.547, 2.751)	0.619
Hypertension	Yes	No	1.043 (0.735, 1.480)	0.815
Heart failure	Yes	No	0.806 (0.259, 2.511)	0.709
Renal failure	Yes	No	0.847 (0.502, 1.429)	0.534
Stroke history	Yes	No	0.582 (0.145, 2.338)	0.446
Asthma	Yes	No	0.733 (0.488, 1.102)	0.136
Pulmonary disease	Yes	No	0.61 (0.228, 1.636)	0.326
Medication	Yes	No	1.178 (0.955, 1.455)	0.126
Anticoagulant	Yes	No	0.333 (0.106, 1.043)	0.059
Immunosuppressant	Yes	No	0.843 (0.269, 2.264)	0.769
Autoimmune disease	Yes	No	1.231 (0.831, 1.823)	0.300
ATC-cardiac	Yes	No	1.001 (0.756, 1.324)	0.997
ATC-pulmonary	Yes	No	0.792 (0.559, 1.124)	0.191
Comorbidities	One	None	1.089 (0.809, 1.468)	0.574
	At least two		0.884 (0.628, 1.242)	0.476

Bold values highlight the significant results with p-value<0.05.

**Figure 4:**
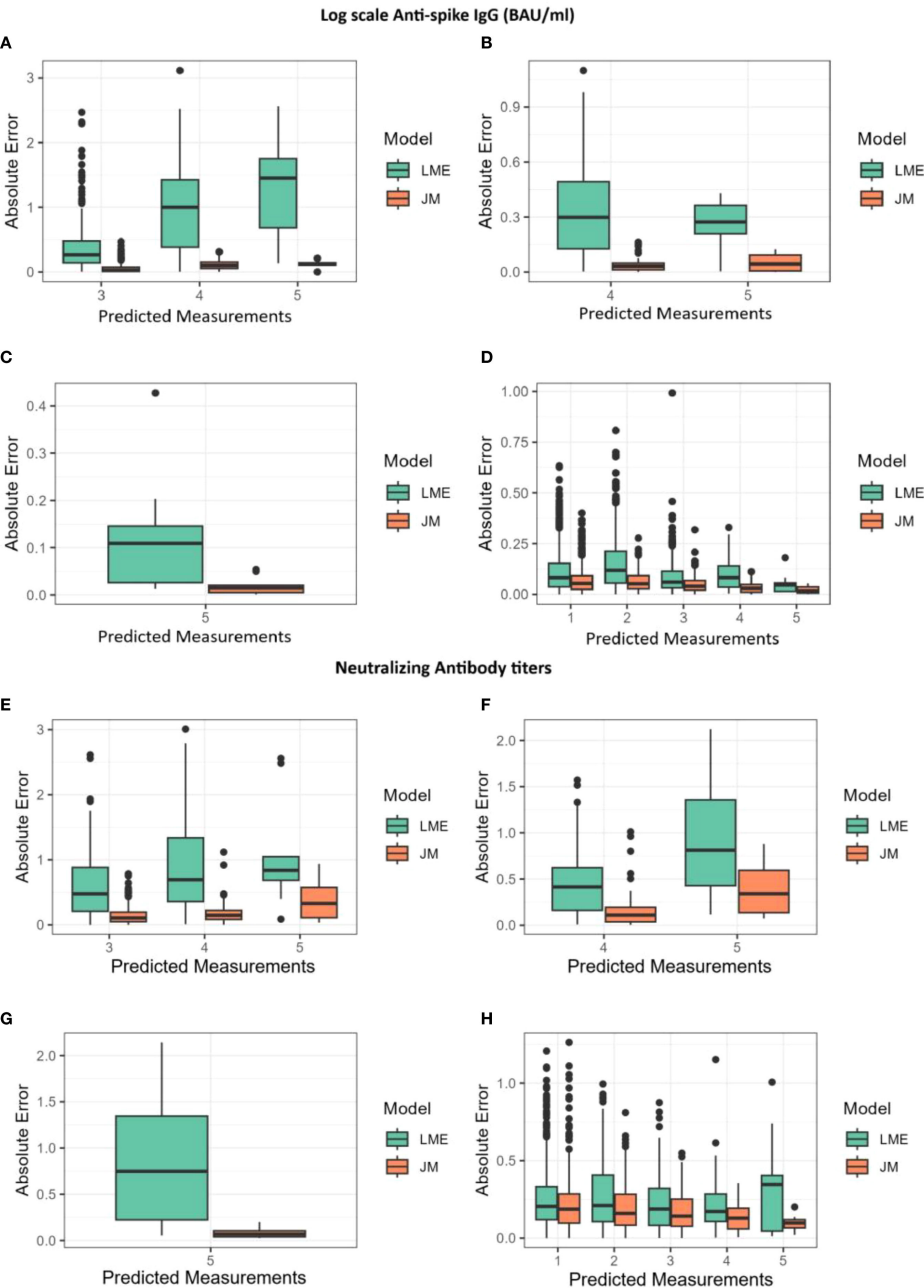
Absolute errors for predictions of log(IgG) and log(NAb) shown alongside the joint model (JM) and the linear mixed-effects (LME) model. The x-axis represents the predicted measurements, while the y-axis shows the absolute error. **(A, E)** Absolute errors for predictions at the third, fourth, and fifth measurements using data from the first two measurements. **(B, F)** Absolute errors for predictions at the fourth and fifth measurements using data from the first three measurements. **(C, G)** Absolute errors for predictions at the fifth measurement using data from the first four measurements. **(D, H)** Absolute errors for predictions at the first, second, third, fourth, and fifth measurements using all available data.

**Table 4:** Longitudinal multivariable models for log-scale neutralizing antibodies and anti-S IgG and survival multivariable models for infection after booster vaccination adjusted for either neutralizing antibodies or anti-S IgG.

	Variables	Category	^1^Longitudinal outcome: neutralizing antibodies		^2^Longitudinal outcome: anti-S IgG	
			Estimate (95% CI)	P-value	Estimate (95% CI)	P-value
Longitudinal multivariable models	**Intercept**		6.919 (6.637, 7.201)	<0.001	9.082 (8.818, 9.345)	<0.001
	**Time (days)**	**ns (time)1**	−2.2 (−2.366, −2.033)	<0.001	−1.921 (−2.041, −1.801)	<0.001
		**ns (time)2**	−2.426 (−2.785, −2.068)	<0.001	−3.29 (−3.56, −3.021)	<0.001
		**ns (time)3**	−2.109 (−2.412, −1.805)		−2.444 (−2.693, −2.196)	
	**Age at booster (years)**		−0.017 (−0.022, −0.013)	**<0.001**	−0.01 (−0.015, −0.006)	<0.001
	**Infection prior to booster**	**No**	**(Ref)**			
		**Yes**	0.204 (0.053, 0.355)	<0.001	–	*-*
	**Infection between primary and booster vaccinations**	**No**			**(Ref)**	
		**Yes**	–	–	0.681 (0.496, 0.865)	<0.001
	**Primary vaccine type**	**Adenovirus vectored**	**(Ref)**		**(Ref)**	
		**mRNA**	0.221 (0.069, 0.373)	<0.001	0.358 (0.207, 0.508)	<0.001

			^3^Survival model adj. for log_e_(NAb)		^4^Survival model adj. for log_e_ (anti-S IgG)	
			HR (95% CI)	p-Value	HR (95% CI)	p-Value
Survival multivariable models	**Age at booster (years)**		0.975 (0.955, 0.995)	0.015	0.974 (0.954, 0.994)	0.012
	**Infection prior to booster**	**No**	**(Ref)**		**(Ref)**	
		**Yes**	0.67 (0.52, 0.88)	0.004	0.62 (0.48, 0.81)	**<0.001**
	**Primary vaccine type**	**Adenovirus vectored**	**(Ref)**		**(Ref)**	
		**mRNA**	0.21 (0.07, 0.63)	0.005	0.19 (0.064, 0.557)	0.003
	**Delay between primary and booster vaccinations (days)**		0.996 (0.992, 0.99)	0.024	0.996 (0.992, 1.00)	0.029
	**Interaction term: age at booster-primary vaccine type mRNA**		1.029 (1.006, 1.053)	0.024	1.032 (1.009, 1.056)	0.007
	**Baseline log_e_ (neutralizing antibodies)**		**0.808 (0.72, 0.90)**	**<0.001**	**-**	**-**
	**Baseline log_e_ (anti-S IgG)**		**-**	**-**	**0.805 (0.72, 0.90)**	**<0.001**

^1^Linear mixed-effects (LME) multivariable model for neutralizing antibody measurements as outcome.

^2^LME multivariable model for anti-S IgG measurements as outcome.

^3^Survival multivariable model adjusted for baseline neutralizing antibody titers (2 weeks).

^4^Survival multivariable model adjusted for baseline anti-S IgG (2 weeks).Bold values highlight the significant results with p-value<0.05.

### Joint modeling of NAb and anti-S IgG with time to SARS-CoV-2 breakthrough infection

Combining the longitudinal data of NAb and anti-S IgG with time to breakthrough infection data under the JM framework allowed us to estimate over time the association between the antibody measurements and the risk of breakthrough infection. At the same time, we derived estimates on longitudinal parameters and adjusted for non-random dropouts. In both JM analyses performed, the declines in the levels of NAb and anti-S IgG over time were associated with increased risk of breakthrough infection (**Table 5**). For log_e_-scaled NAb, a one-unit increment was associated with 27.0% lower risk of breakthrough infection (HR, 95% CI, 0.73, [0.63 to 0.83], p< 0.001). For log_e_-scaled anti-S IgG, one-unit increment was associated with 22.0% lower risk of breakthrough infection (HR, 95% CI, 0.78, [0.70 to 0.87], p< 0.001). In both survival sub-models, older ages at the date of the booster dose, infection prior to booster, and primary vaccination with mRNA vaccines (*vs*. adenovirus-vectored vaccines) were shown to be protective factors against a breakthrough infection. More specifically, for every 1-year increase in age, the risk of a breakthrough infection decreased by 0.3% (HR, 95% CI, 0.997, [0.995 to 0.999], p = 0.022) in both JMs. Participants infected prior to booster vaccination presented 26% (HR, 95% CI, 0.740, [0.560 to 0.970], p = 0.030) lower risk of breakthrough infection in the NAb-based JM and 34% (HR, 95% CI, 0.660, [0.50 to 0.840], p = 0.001) lower risk in the anti-S IgG-based JM. Participants primary-vaccinated with mRNA-based vaccines experienced a 79% lower risk of infection in both JMs. At the same time, the longitudinal values of NAb and anti-S IgG waned as time passed and age increased. For a longer delay between the primary and booster vaccinations, JM analysis showed that both levels of NAb and anti-S IgG increased.

**Table 5:** Longitudinal and survival sub-models for neutralizing antibodies and anti-S IgG for infection after booster vaccination under joint modeling framework*.

Variables	Category	Neutralizing antibodies (joint model 1)		Anti-s IgG (joint model 2)	
		^1^Longitudinal sub-model		^2^Longitudinal sub-model	
		Estimate (95% CI)	P-value	Estimate (95% CI)	P-value
Intercept		6.78 (6.51, 7.06)	<0.001	9.08 (8.82, 9.34)	<0.001
Time (days)	**ns (time)1**	−2.13 (−2.29,−1.97)	<0.001	−1.87 (−1.98, −1.75)	<0.001
	**ns (time)2**	−2.41 (−2.78, −2.06)	<0.001	−3.27 (−3.54, −3.00)	<0.001
	**ns (time)3**	−2.13 (−2.42, −1.85)		−2.44 (−2.66, −2.21)	
Age at booster (years)		−0.016 (−0.02, −0.012)	<0.001	−0.010 (−0.015, −0.006)	<0.001
Infection prior to booster	**No**	**(Ref)**			
	**Yes**	0.49 (0.37, 0.61)	<0.001		
Infection between primary and booster vaccinations	**No**				
	**Yes**			0.68 (0.46, 0.86)	<0.001
Primary vaccine type	**Adenovirus vectored**	**(Ref)**			
	**mRNA**	0.21 (0.06, 0.35)	<0.001	0.36 (0.21, 0.51)	<0.001

		^3^Survival sub-model		^4^Survival sub-model	
		HR (95% CI)	p-Value	HR (95% CI)	p-Value
Age at booster (years)		0.97 (0.95, 0.99)	0.022	0.97 (0.95, 0.99)	0.025
Infection prior to booster	**No**	**(Ref)**			
	**Yes**	0.74 (0.56, 0.97)	0.030	0.66 (0.50, 0.84)	0.0011
Primary vaccine type	**Adenovirus vectored**	**(Ref)**			
	**mRNA**	0.21 (0.066, 0.78)	0.023	0.22 (0.072, 0.65)	0.0043
Delay between primary and booster vaccinations (days)		0.996 (0.993, 1.00)	0.028	0.996 (0.993, 1.00)	0.045
Interaction term: age at booster-primary vaccine type mRNA		1.029 (1.002, 1.056)	0.037	1.029 (1.006, 1.055)	0.010
^5^α		**0.73 (0.63, 0.83)**	**<0.001**	**0.77 (0.70, 0.86)**	**<0.001**

^*^Results of two joint models for time to SARS-CoV-2 breakthrough infection using log-scale neutralizing antibody and log-scale anti-S IgG measurements as the longitudinal response each time.

^1^Linear mixed-effects (LME) model for neutralizing antibody measurements as outcome.

^2^LME model for anti-S IgG measurements as outcome.

^3^Survival sub-model of the joint model with neutralizing antibody titers trajectories.

^4^Survival sub-model of the joint model with anti-S IgG trajectories.

^5^Neutralizing antibody titers estimate for joint model 1 and anti-S IgG estimate for joint model 2.Bold values highlight the significant results with p-value<0.05.

### Cross-validation of prediction performances and visualization of participant-specific dynamic prediction

Finally, to compare the different modeling strategies, both JMs and longitudinal models were submitted to a 10-fold cross-validation process as described in the Materials and Methods (**Supplementary Figure S7**). Their overall performances were assessed by calculating the mean absolute difference [absolute error (AE)] between the predicted and observed log(IgG) and log(NAb) measurements. AEs in function of the number of measurements used for predictions are shown in **Figure 4**, while a detailed summary alongside the interquartile range (Q1–Q3) is provided in **Supplementary Table S1**. Throughout the follow-up period, AEs remained consistently low for both JMs and decreased as additional measurements became available. The JMs consistently produced lower prediction errors than the LME model (**Figure 4**). In JMs with log(NAb) as the outcome of the longitudinal sub-model, errors were approximately 19% smaller than the errors of the LME model (median AE, 0.17 *vs*. 0.21), with RMSE values confirming this trend (0.27 *vs*. 0.32). In JMs with log(IgG) as the outcome of the longitudinal sub-model, the difference was even more pronounced, with AEs from the JM being nearly half the size of those from LME models (median AE, 0.05 *vs*. 0.09; RMSE, 0.08 *vs*. 0.16). The greatest improvement was observed for predictions of the fifth measurements, where RMSE from JMs was reduced by a factor of more than five in some cases. These results demonstrate that JM provides more accurate predictions.

**Figure 5:**
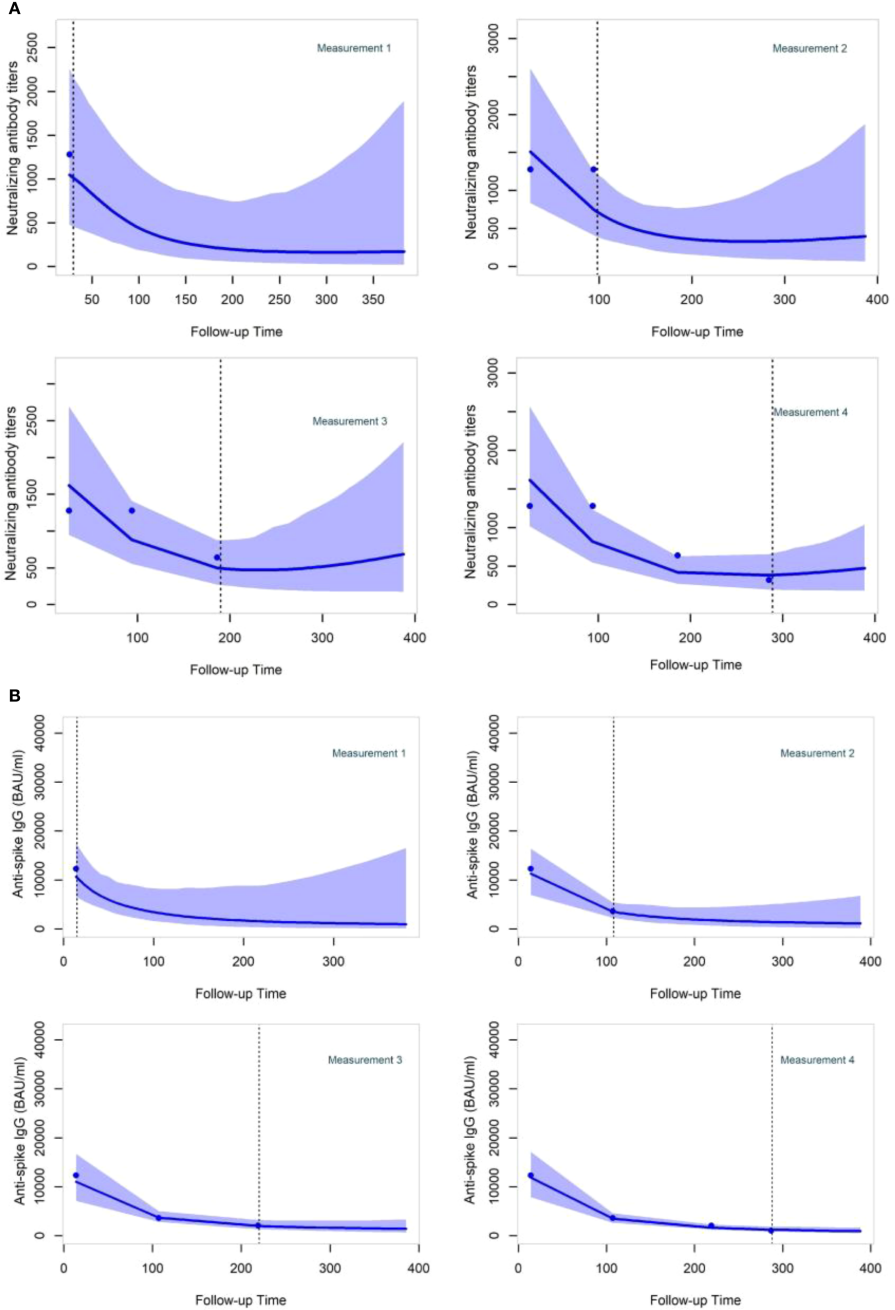
Neutralizing antibody titers and anti-Spike IgG dynamic predictions for two specific participants **(A, B)** after the booster dose administration. Participant **(A)** was a 59-year-old man who received the primary vaccination with BNT162b2. He underwent a booster shot with BNT162b2–193 days after the initial vaccination and had not been previously infected before receiving the booster dose. Participant **(B)** was a 63-year-old man who received the primary vaccination with ChAdOx1-S. He underwent a booster shot with BNT162b2–144 days after the initial vaccination and had not been previously infected before receiving the booster dose.

As examples, participant-specific dynamic predictions for NAb and anti-S IgG in participants with different vaccinations and prior infection profiles are visualized, respectively, over four panels representing a longitudinal follow-up where successive measurements become available for a patient (one to four measurements) after the booster vaccination (**Figure 5**). On each panel, the left side reflects the observed values of NAb or anti-S IgG, represented by dots. The vertical dashed line represents the present time, the right side depicts the prediction estimation, and the shaded area shows the 95% confidence interval of the predictions. Both JMs can update predictions at each time point as new information on NAb or anti-S IgG becomes available. The 95% confidence interval of the predictions becomes shorter with the addition of more longitudinal information, thereby strengthening the accuracy of the predictions.

## Discussion

In this prospective, longitudinal study, we present data from the follow-up of 966 participants after the booster vaccination against SARS-CoV-2. Unlike most other studies, we not only identified the factors associated with variations in humoral responses but also placed these measurements alongside the monitoring of infection occurrence carried out throughout the same period. Then, on the basis of these observations, we established and compared different statistical models, enabling us to make personalized predictions of the evolution of the anti-viral immune response over time. The results generated provide important information for targeted vaccination of vulnerable populations, e.g., immunocompromised or older people, or those for whom it is important to limit their contamination and therefore their power of transmission, e.g., healthcare workers.

In relation to the waning of antibodies after the booster dose, both NAb and anti-S IgG antibodies appeared to decline at a similar rate. Our study supports findings from others, indicating that older individuals tend to produce fewer NAb and anti-S IgG, while those with a higher BMI exhibit higher levels of NAb and anti-S IgG (49, 50). Previous studies (51–53) have underscored the effect of prior infection and the usage of mRNA as primary vaccines and mRNA booster vaccination in preventing infections. Our study, in line with these observations, shows that infection before booster, after primary vaccination and not before mRNA-based primary vaccinations, and the usage of mRNA-1273 as booster vaccine resulted in higher antibody levels. Interestingly, we observed a significant positive correlation between the delay between the primary and booster vaccinations and the levels of both NAb and anti-S IgG. This result highlights the necessity of considering the optimal timing for administering a booster. Future studies with longer follow-ups and more repeated measurements of antibody levels will be necessary to further investigate this matter.

In investigating the risk factors for infection following the booster dose, we conducted a univariable analysis based on Cox-PH models, considering various variables related to individual characteristics, vaccination history, antigen exposures, and comorbidities. Consistent with previous studies (15, 21), our study reveals that infection before the boost significantly reduces the risk of subsequent infection after receiving the booster dose. However, it was only the case when infection occurred between the primary and booster vaccinations. This result is in line with our previous results showing that hybrid immunity was only better if the first exposure to SARS-CoV-2 antigens was through vaccination, not infection (47). Taking into consideration that the last primary vaccination for the participants occurred in early September 2021, 3 months before the dominance of the Omicron variant, our results could reflect the limited protection offered by the primary vaccination and pre-primary infection against this variant (54).

Through the longitudinal collection of blood samples after the primary vaccination, we were able to assess the relationship between the levels of NAb and anti-S IgG before administering the booster and the risk of subsequent breakthrough infection. Our findings indicate that higher pre-booster levels of NAb and anti-S IgG were associated with a lower risk of infection after the booster dose.

In contrast to previous studies (15), we observed that elevated BMI was correlated with a lower risk of infection. This result is consistent with the association of higher BMI and increased antibody levels, also observed by other groups (55) and in our previous study (47), where BMI was positively correlated with levels of NAb and anti-S IgG after two antigen exposures. Importantly, no particular vaccination program was applied to people with higher BMI. We also did not observe differences in waning of immunity in contrast to what was observed in another cohort, where people with severe obesity (BMI > 40) displayed accelerated waning of humoral immunity (56). Our results also revealed that participants who received mRNA vaccines as primary vaccination had a lower risk of infection after the booster compared to those vaccinated with viral vector vaccines. Specifically, individuals vaccinated with BNT162b2 and mRNA-1273 displayed a reduced risk of infection compared to those who received ChAdOx1-S and Ad26.COV2.S (57). Furthermore, mRNA primary vaccination followed by an mRNA booster proved to be a more protective factor against breakthrough infections compared to adenovirus-based primary vaccination followed by an mRNA booster. The increased delay between the primary vaccination and the booster also reduced the risk of breakthrough infections, suggesting again that there is an optimal timing for boosting if we only look at protection after the boost. Finally, none of the comorbidities or diseases we investigated were shown to be significant risk factors for breakthrough infections, as previously shown (15). The demographic characteristics of our cohort, a university-based population of relatively young age, were likely fundamental factors leading to these results, as our cohort, in general, is likely representative of a healthy population.

We assessed the association between repeated measurements of NAb and anti-S IgG with breakthrough infections following booster vaccination. To the best of our knowledge, our study is among the first to investigate the risk of infection after the booster dose in non-hospitalized individuals, utilizing the underlying waning trajectories of NAb and anti-S IgG through the implementation of a joint modeling design. Another study in the Netherlands (58) used this design for COVID-19 patients but focusing on various biomarkers and predicting mortality in patients admitted to the intensive care unit (ICU). Considering a design employing joint modeling (38) may prove beneficial in investigating the longitudinal marker’s capability to characterize the time-to-event endpoint. This is provided that the sub-model’s assumptions are correctly specified. Joint models represent a powerful tool with demonstrated higher performance over the alternative approach of the time-dependent Cox-PH model (59). We present two univariable joint models, one for each of the longitudinally collected outcomes. Because of the high correlation between NAb and anti-S IgG, applying a multivariable joint model containing both was deemed impossible. Participants who were previously infected and received primary vaccination with mRNA vaccines exhibited the highest levels of NAb and anti-S IgG, while older individuals showed lower levels. Higher longitudinal measures of NAb and anti-S IgG were highly associated with a decreased risk of post-booster breakthrough infection. These findings provide risk estimates taking into account the evolution of the repeated measurements based on the JM design. We were able to show a significant risk reduction associated with increasing age, despite the lack of significance observed in the univariable Cox-PH model. This finding can be attributed to the inclusion of the significant interaction term between age and the type of primary vaccination, reflecting that older participants were prioritized to receive mRNA vaccines earlier due to the potential risk associated with adenovirus-vectored vaccines. Infection prior to booster, mRNA primary-vaccinated participants, and the delay between the primary and booster vaccinations remained significant risk factors in the JM analysis, revealing a lower risk for breakthrough infections.

The major strength of this study is its large sample size, considering the amount of longitudinally collected information, especially the repeated saliva testing for infection and the scheduled blood sampling. Considering our previous study (47) on the same cohort, which highlighted some factors driving NAb and anti-S IgG levels according to the number and sequence of antigen exposures, this study provided a clear analysis of breakthrough infection risk factors within boosted populations. Importantly, the use of joint models is able to provide useful insights, which can be used by healthcare professionals to visualize real-time personalized antibody predictions at any time during a follow-up and at every time they obtain a measurement. In the future, a longer follow-up with more repeated measurements, a bigger sample size, and more informative JM using time-dependent covariates in the survival sub-model analysis (60), such as the virus circulating and socializing data, could also provide more accurate predictions for survival probabilities concerning personalized future infection.

There are also some limitations that need to be mentioned. Assumption checks for the normality of conditional residuals and random effects were performed using empirical Bayes estimates. While this approach is widely applied, it is known to be only an approximation and may not fully capture departures from normality (42). Therefore, the diagnostic results should be interpreted with caution. The incorporation of the time-to-event sub-model allows us to reduce bias arising from informative dropout since antibody measurements are jointly modeled with the risk of infection (44). However, this correction depends on the joint model being correctly specified and may not fully eliminate bias under more complex missingness mechanisms. Although anti-N analysis was a strong tool to detect subclinical infections during the follow-up, we were unable to determine the date of infection, which is necessary to compute the time from booster to infection for survival models and sub-models in JM analysis. In order to avoid excluding participants for whom the infection date could not be determined, we excluded the blood samples that showed positivity to anti-N while infection was not reported by another method. Consequently, we followed up these participants (n = 24) until the last available information when we knew they were uninfected, which was the date of the last blood sample showing a negative anti-N serology. However, this procedure could have introduced bias into our analysis. Another limitation in our study is the lack of socializing contact information. We hypothesize that the reduced risk of infection among older participants could be explained by reduced social activity compared to younger participants in our cohort. Similarly, people with higher BMI could also be more cautious because of the increased risk of severe disease, which could explain why increased BMI is associated with lower infection in our study. Unfortunately, no data have been collected to address these questions. Some other confounding factors may also exist. In Belgium, boosters were initially offered to specific high-risk categories and then expanded to the broader population to ensure optimal immunity across the population, especially for vulnerable groups and older adults. Thus, people primarily vaccinated with viral vector vaccines were prioritized for booster doses ahead of those who had received mRNA vaccines. This decision was due to emerging data suggesting that immunity from viral vector vaccines may wane faster than immunity from mRNA vaccines, making additional protection important sooner for those primarily vaccinated with viral vector vaccines.

One use of JM is to obtain dynamic predictions focusing on the longitudinal outcome while correcting for non-random dropout due to breakthrough infection events. Another use is to obtain dynamic, or evolving, risk predictions. In addition to individualized predictions of antibody levels, we also derived dynamic survival predictions where updated antibody trajectories were used to estimate the risk of breakthrough infection. The predictive performance of these models, evaluated using time-dependent Area Under the Curve (AUC), sensitivity, and specificity, was moderate (**Supplementary Table S2** and **Supplementary Figure S14**). This moderate performance reflects the fact that infection risk is strongly influenced by external factors such as virus circulation, variant prevalence, and individual social contacts, which were not included in our models. Nevertheless, by leveraging longitudinal antibody measurements through joint modeling, we demonstrate the feasibility of generating individualized predictions of both antibody dynamics and infection risk. These dynamic predictions can be considered a first step toward a more personalized approach to booster vaccination, where future models incorporating additional data (e.g., social contacts, cellular immunity, and virus circulation) may provide improved predictive accuracy and greater clinical utility. Therefore, while the joint models provide individualized risk estimates, these should be interpreted with caution. Such an approach could ultimately help guide individualized vaccination strategies, for example, in vulnerable populations or in critical healthcare personnel where infection prevention is important. In conclusion, based on data from the field, we proved that JMs are a valuable tool for analyzing longitudinal measures and provide accurate predictions of immune response trajectories. To predict the risk of infection accurately in the future, more extensive data, including longer follow-ups, are essential. This should encompass information on social contacts, exogenous factors such as regional infection incidence, and a more comprehensive recording of longitudinal markers. Moreover, predicting the risk of infection in the general population would also require including other population strata, as we focused here on a rather “healthy” population, as stated before. Indeed, despite the non-significant results regarding comorbidities, numerous studies have shed light on social groups with comorbidities being more vulnerable not only to infection but also to severe outcomes. The decline in protection varies among different individual profiles. This variation raises questions about the optimal timing for boosting. As countries have lifted restrictions related to public health, we find ourselves in an era of personal responsibility for hygiene and social contact. While general population vaccination campaigns, whether before or during SARS-CoV-2 waves, represent a reasonable strategy to maintain humoral immunity across the masses, it is equally important for research and public health policies to incorporate personalized decision-making. The models developed in this study could help reach this goal in the context of SARS-CoV-2 but also for any other infectious disease that would require it.

## Data Availability

The raw data supporting the conclusions of this article will be made available by the authors, without undue reservation.
