# Supplementary material for: Personalized prediction of SARS-CoV-2 vaccine-induced immunity after boost: a longitudinal analysis using joint modeling
Source: Front Immunol. 2025 Sep 30;16:1619631. doi: 10.3389/fimmu.2025.1619631 (PMC12518370; doi:10.3389/fimmu.2025.1619631)
Supplement: Supplementary file 1 [file DataSheet1.pdf]

# Personalized Prediction of SARS-CoV-2 Vaccine-Induced Immunity after Boost: A Longitudinal Analysis Using Joint Modelling

Papadopoulos, I.[1], Diep, A.N.[1], Schyns, J.[2, 3], Gourzones, C.[3, 4], Minner, F.[3], Bonhomme, G.[5], Paridans, M.[6], Gillain, N.[1,6], Husson, E. [1,6], Garigliany, M. [5], Darcis, G.[7], Desmecht, D. [5], Guillaume, M.[6,] , Bureau, F.[2, 3, *], Donneau, A.F.[1, *], Gillet, L.[3, 4, *].

1 Biostatistics and Research Method Center - Public Health Department, Liège University, 4000 Liège, Belgium
2 Laboratory of Cellular and Molecular Immunology, GIGA Institute, Liège University, 4000 Liège, Belgium
3 COVID-19 platform, Liège University, 4000 Liège, Belgium
4 Laboratory of Immunology-Vaccinology, FARAH, Liège University, 4000 Liège, Belgium
5 Department of Pathology, FARAH, Liège University, 4000 Liège, Belgium
6 From Biostatistics to Health Promotion Research Unit, Public Health Department, Liège University, 4000 Liège, Belgium
7 Infectious Diseases Department, Centre hospitalier universitaire de Liège, 4000 Liège, Belgium

* co-senior authors, correspondence: fabrice.bureau@uliege.be, afdonneau@uliege.be, L.gillet@uliege.be

## Supplementary Material

Overview: This supplementary material contains some additional informative plots and tables that were not included in the main manuscript and plots that deal with modelling diagnostics. In the presented plots, we provide the Flow diagram for the eligibility criteria applied to conclude the final sample (**Fig. S1**). We describe the visiting process during which we collected blood samples in the follow-up after the booster vaccination for the final participant sample included in our analysis (**Fig. S2**). A multi-plot (**Fig. S3**), similar to Fig. 2 included in the main manuscript, highlights temporal changes by inserting a Loess smoother to emphasize the slightly non-linear relation through time. **Fig. S4** shows longitudinal changes over time by different vaccines alongside density plots to examine distribution differences. Longitudinal differences between some of the variables that were found to be non-significant in univariable analysis are presented in **Fig. S5**. Kaplan-Meier plots for twelve variables that were not included in the multivariable survival analysis are presented (**Fig. S6**). A diagram of describing the process of a 10-fold cross validation of predictions obtained from JM models (**Fig. S7**). Diagnostic plots for the multivariable survival sub-model are presented in Part 1.2, including Schoenfeld, Martingale, and Cox-Snell residuals (**Fig. S8-S9**). Based on these resulting plots, we conclude a high model performance. In Part 1.3, diagnostic plots for the longitudinal sub-models of the Joint modelling analysis are presented. **Fig. S10** describes how we treated the variable of time, capturing non-linearity by assuming natural cubic splines with 3 degrees of freedom. **Fig. S11-S12** present the histograms of the residuals and the plots between standardized residuals-fitted values and observed values-predicted for both longitudinal outcomes of Nab and anti-S IgG. The last part, 1.4, contains the density and trace plots (**Fig. S13**) for the 5 chains for both Joint models. **Table S1** presents the median absolute errors with the corresponding 25$^{th}$ and 75$^{th}$ percentiles and root mean square errors for the prediction in **fig. 4(d)** and **fig. 4(h)**.

# Table of Contents

1. Additional Plots ........................................................................................................ 2
1.1 Informative Figures ................................................................................................ 2
1.2 Accessing the performance of the Survival sub-model. ............................................... 9
1.3 Accessing the performance of the Longitudinal sub-model. ....................................... 11
1.4. Accessing the performance of the two Joint models. ................................................ 13

# 1. Additional Plots

## 1.1 Informative Figures

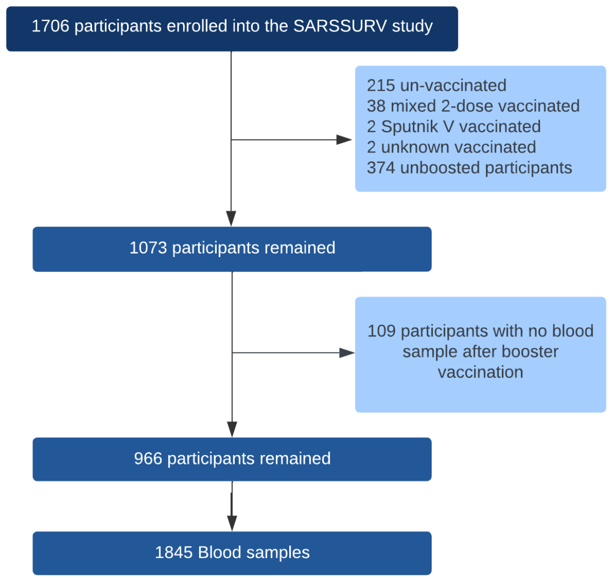


**Fig. S1: Flow Diagram of final study's participants in the analysis.**

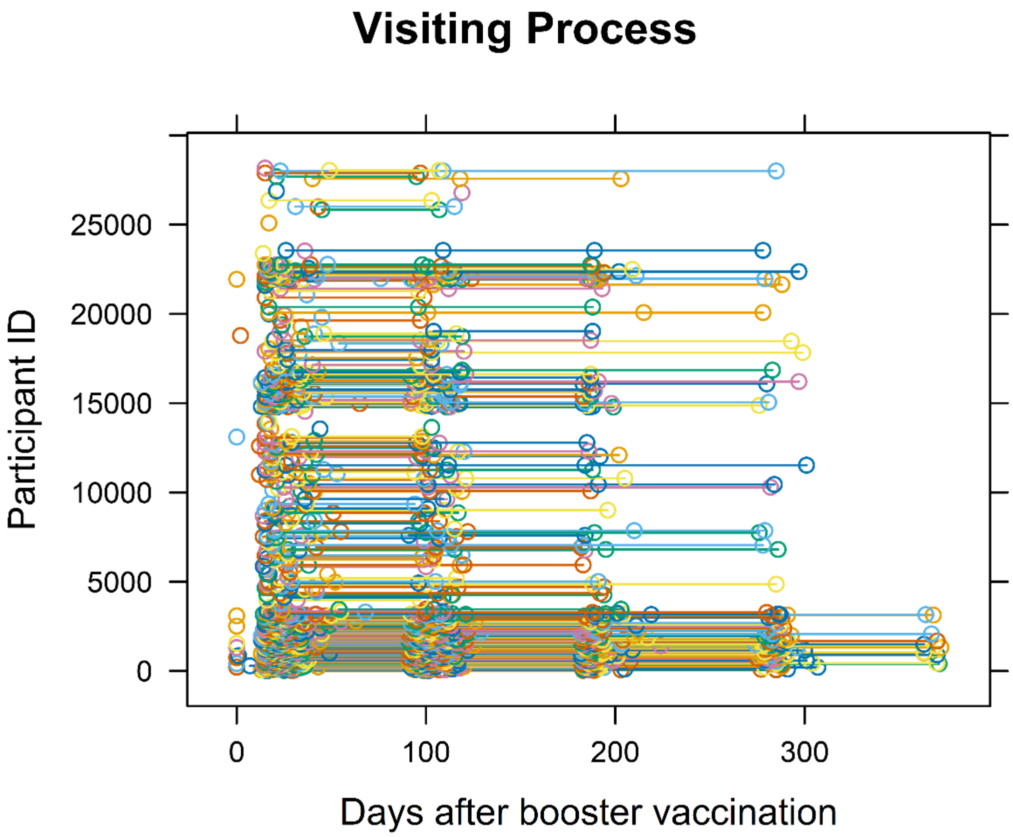


**Fig. S2:** Visiting process for blood sampling after the booster.

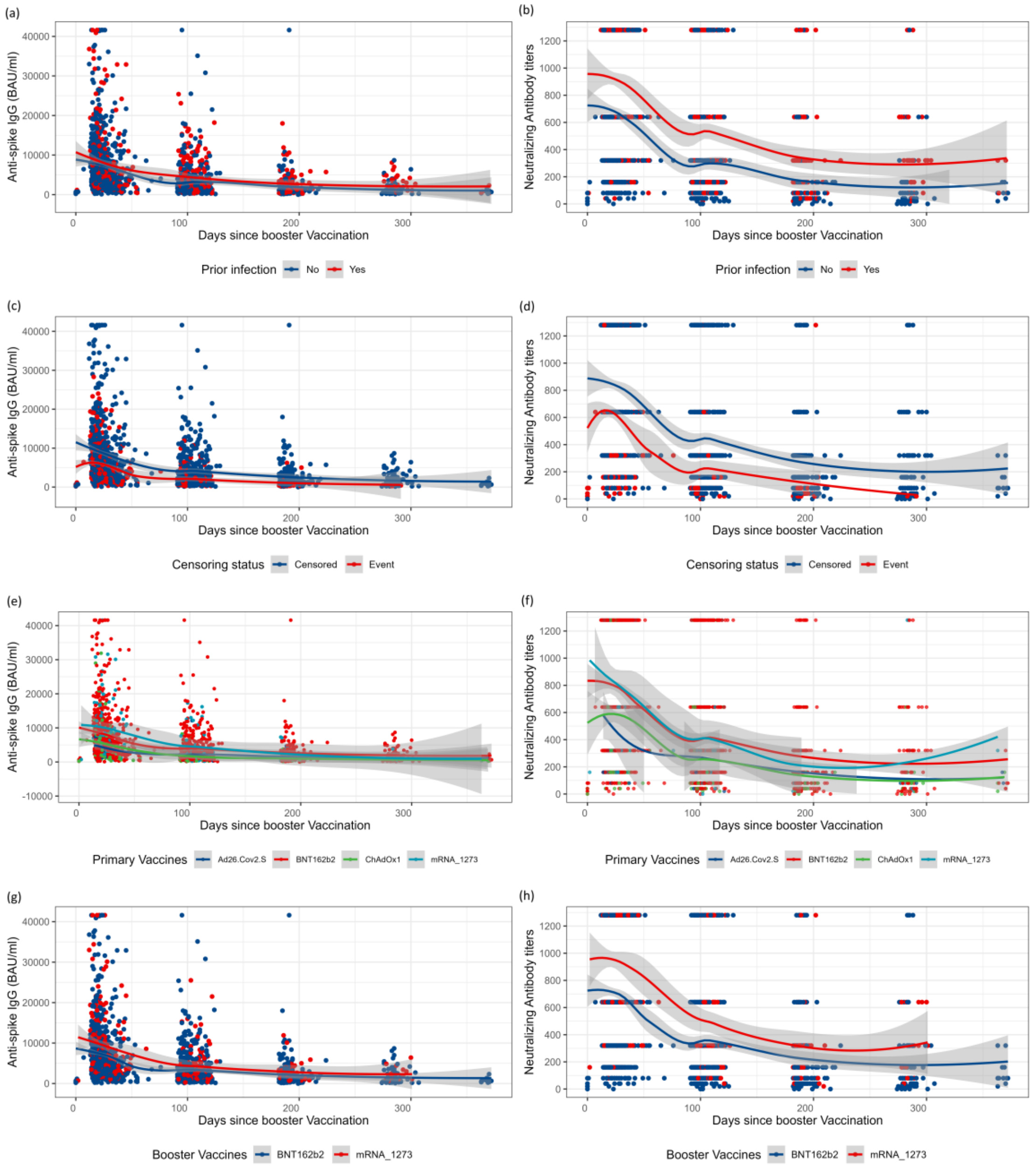


**Fig. S3:** Overall longitudinal changes after the booster vaccination of Neutralizing Antibodies and Anti-S IgG (loess). **(a-b)** Anti-S IgG and Nab values after the booster dose by prior to booster infection status. **(c-d)** Anti-S IgG and Nab values after the booster dose of participants who were infected or not after the booster dose. **(e-f)** Anti-S IgG and Nab values after the booster dose by primary vaccination vaccine types. **(g-h)** Anti-S IgG and Nab values after the booster dose by booster type. The shaded areas represent the 95% confidence interval.

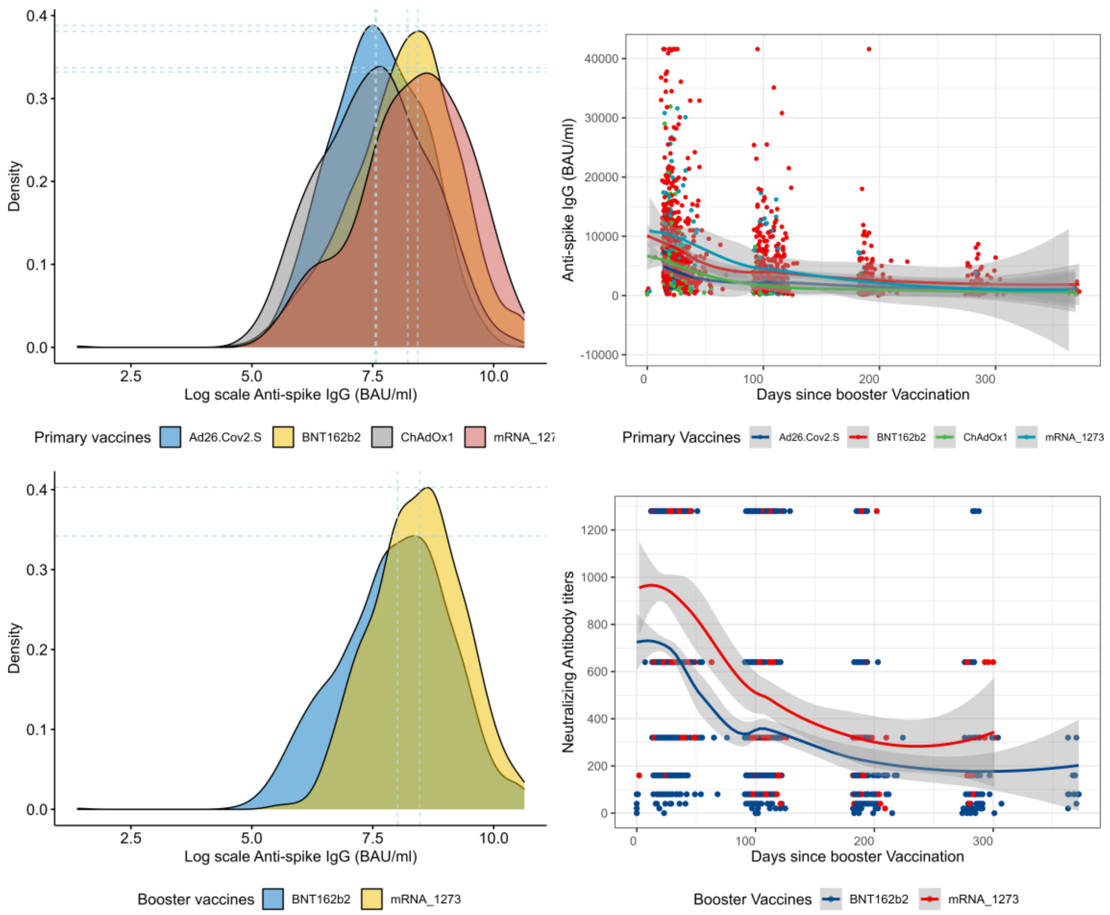


**Fig. S4:** Density plots and temporal waning between primary and booster vaccines.

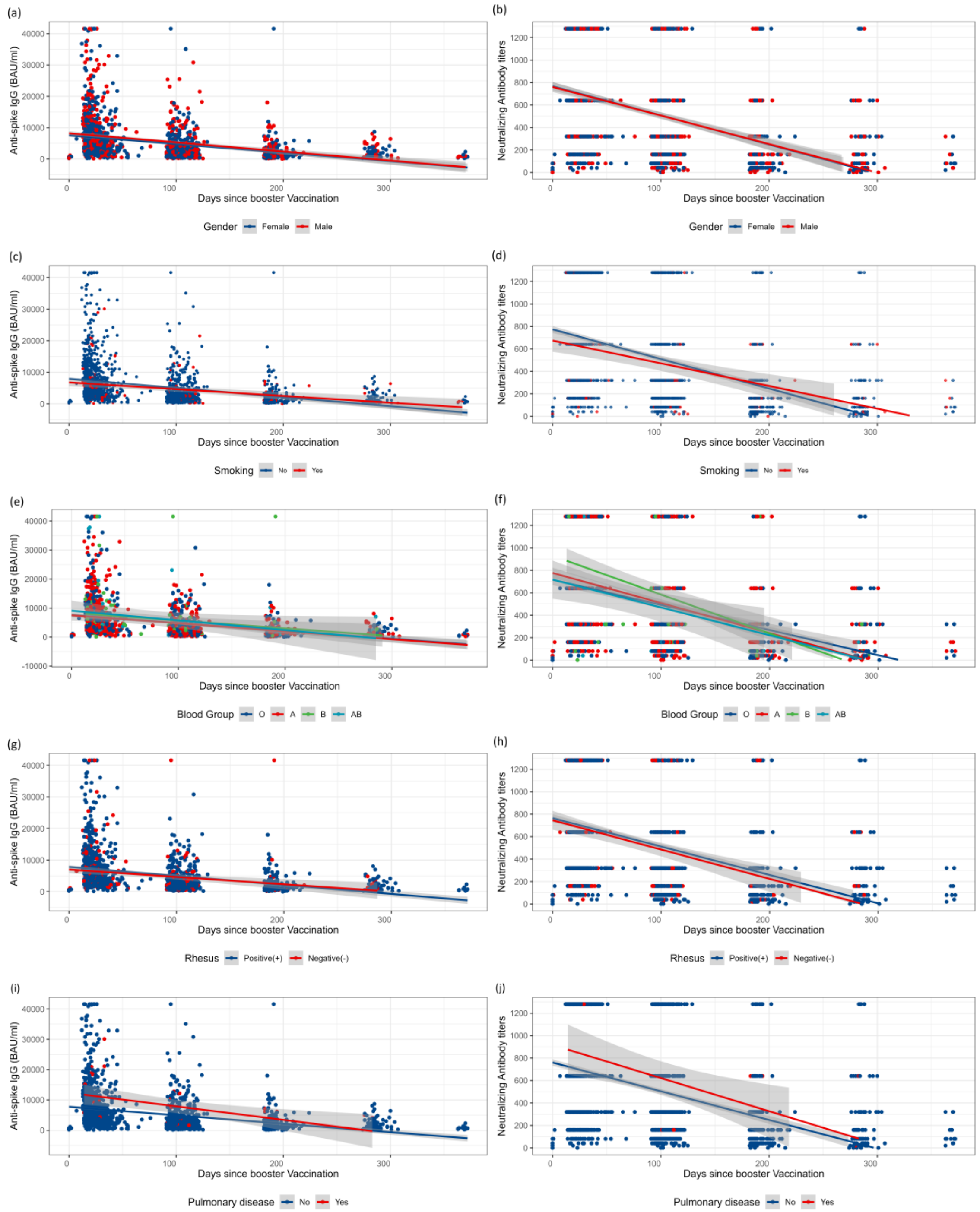


**Fig. S5: Overall longitudinal changes after the booster vaccination of Neutralizing Antibodies and Anti-S IgG.** By column order: **(a-b),** Gender. **(c- d),** Smoking. **(e-f),** Blood Group. (**g-h),** Rhesus group. **(i-j),** Pulmonary Disease.

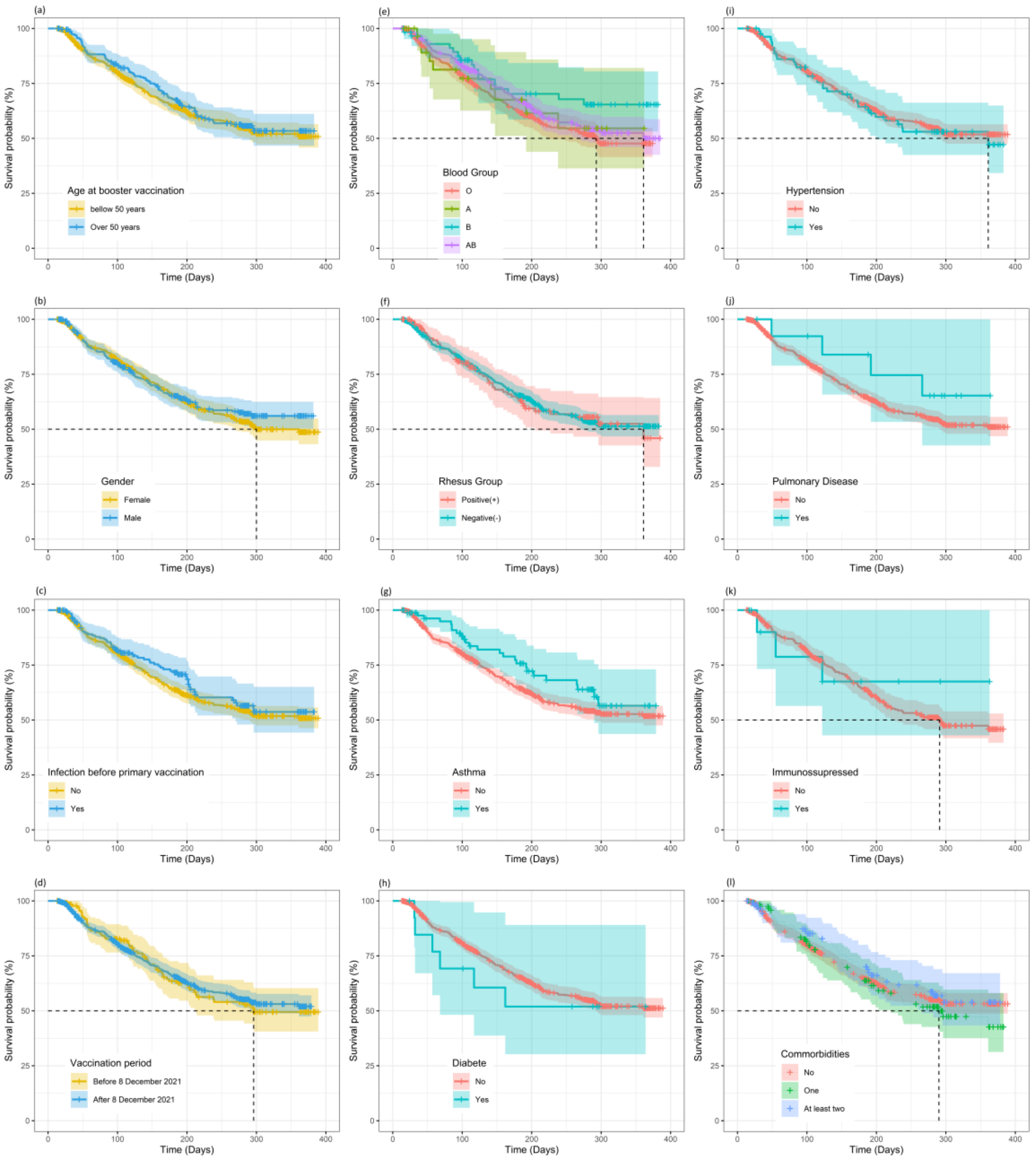


**Fig. S6: Kaplan-Meier plots for twelve variables that were not included in multivariable analysis.** By column order: **(a),** Age at vaccination. **(b),** Gender. **(c),** Infection before primary vaccination. **(d),** Vaccination Period. **(e),** Blood Group. **(f),** Rhesus group. **(g),** Asthma. **(h),** Diabetes. **(i),** Hypertension. **(j),** Pulmonary Disease. **(k),** Immunosuppressed status. **(l),** Comorbidities.

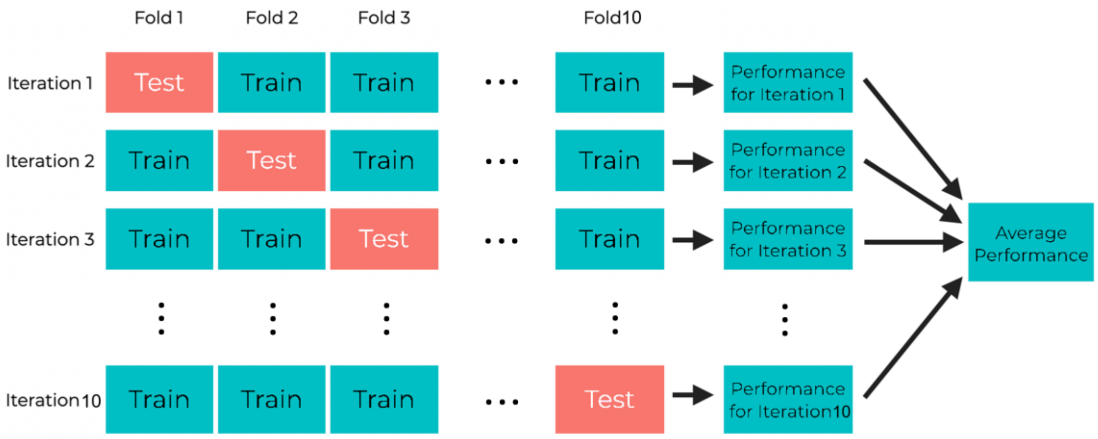


**Fig. S7: Diagram of 10-fold cross validation process.**

## 1.2 Accessing the performance of the Survival sub-model.

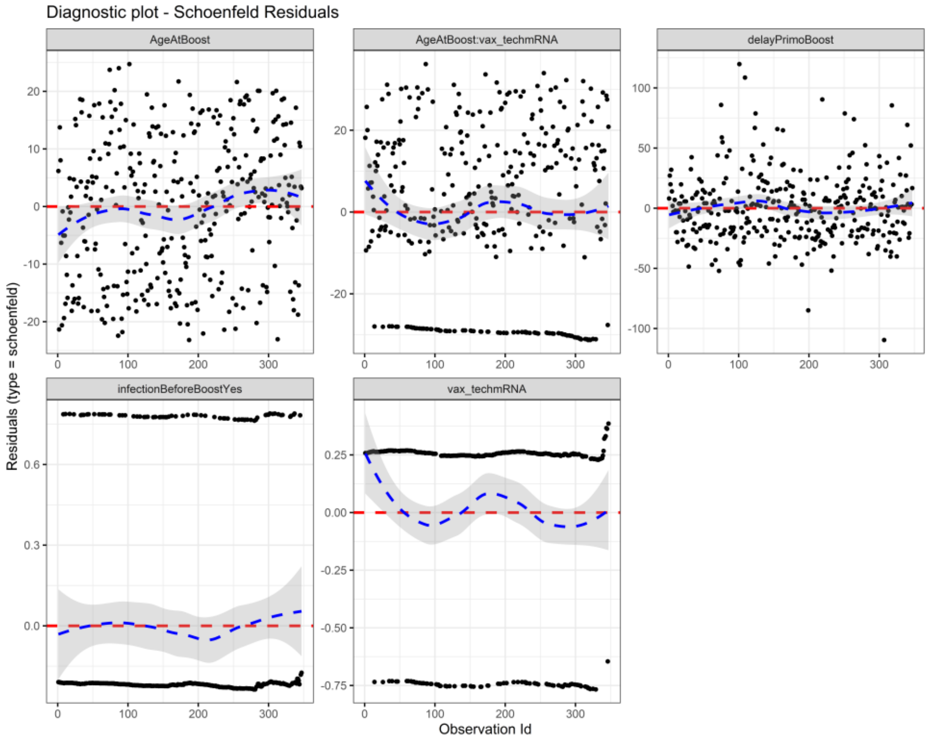


**Fig. S8:** Schoenfeld residuals for the multivariable survival sub-model.

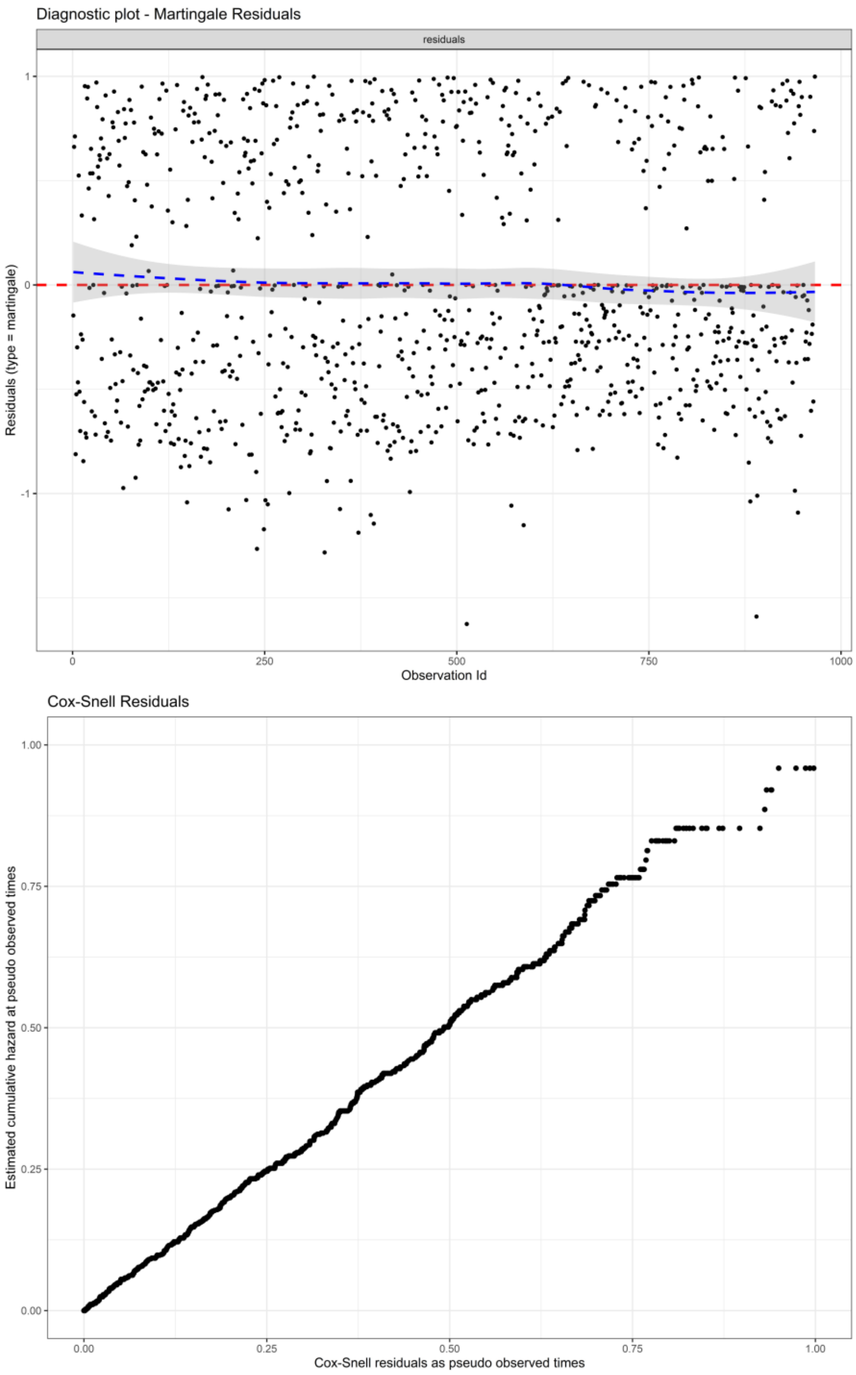


**Fig. S9:** Martingale and Cox-Snell residuals for the multivariable survival sub-model.

## 1.3 Accessing the performance of the Longitudinal sub-model.

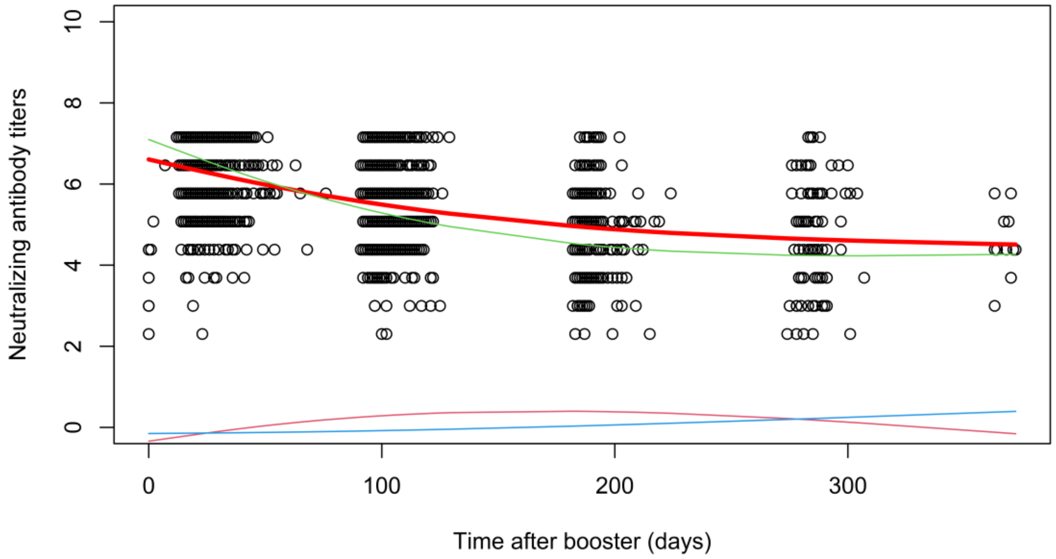


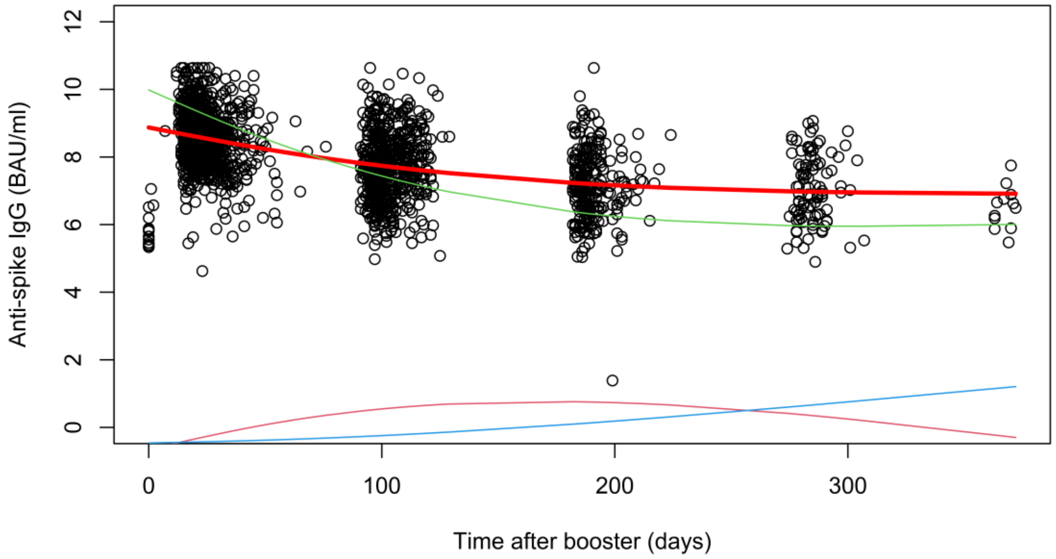


**Fig. S10:** Regression spline function and basis function for Nab and anti-S IgG.

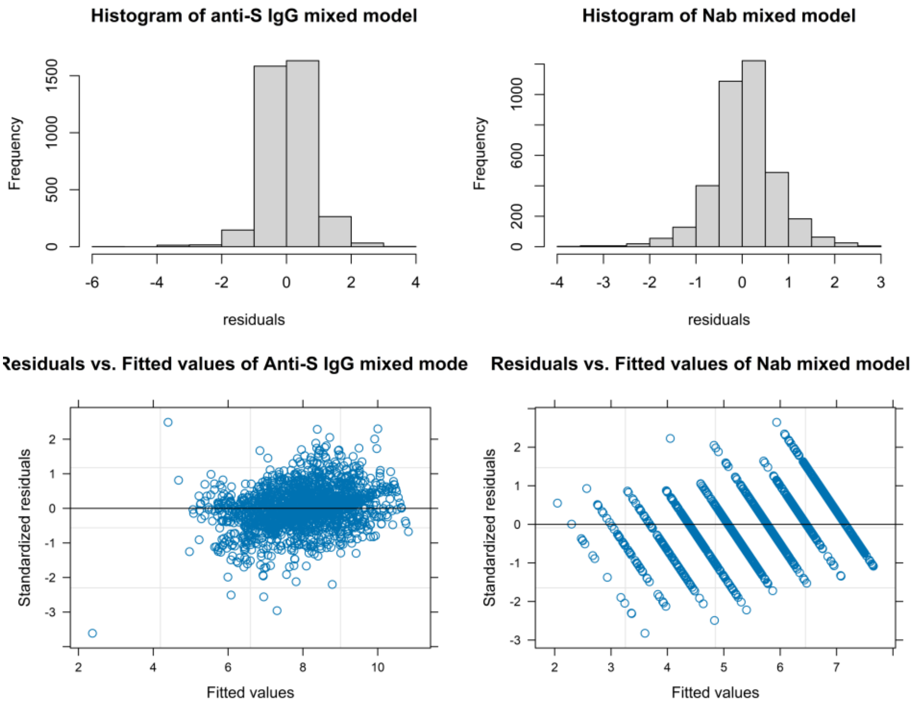


**Fig. S11.** Histogram of residuals of the two longitudinal sub-models.

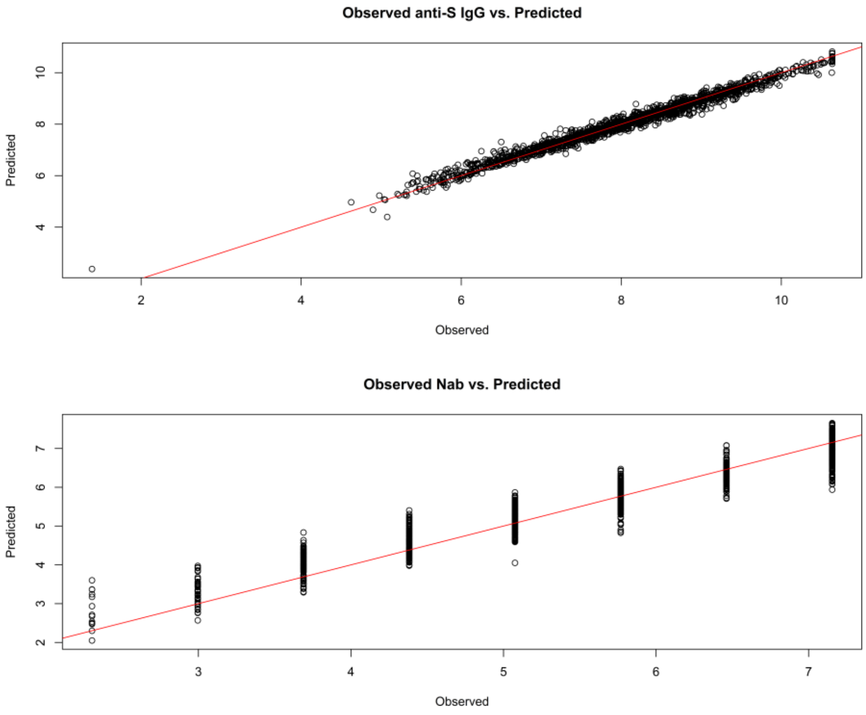


**Fig. S12:** Observed Nab and anti-S IgG values vs. predicted.

## 1.4. Accessing the performance of the two Joint models.

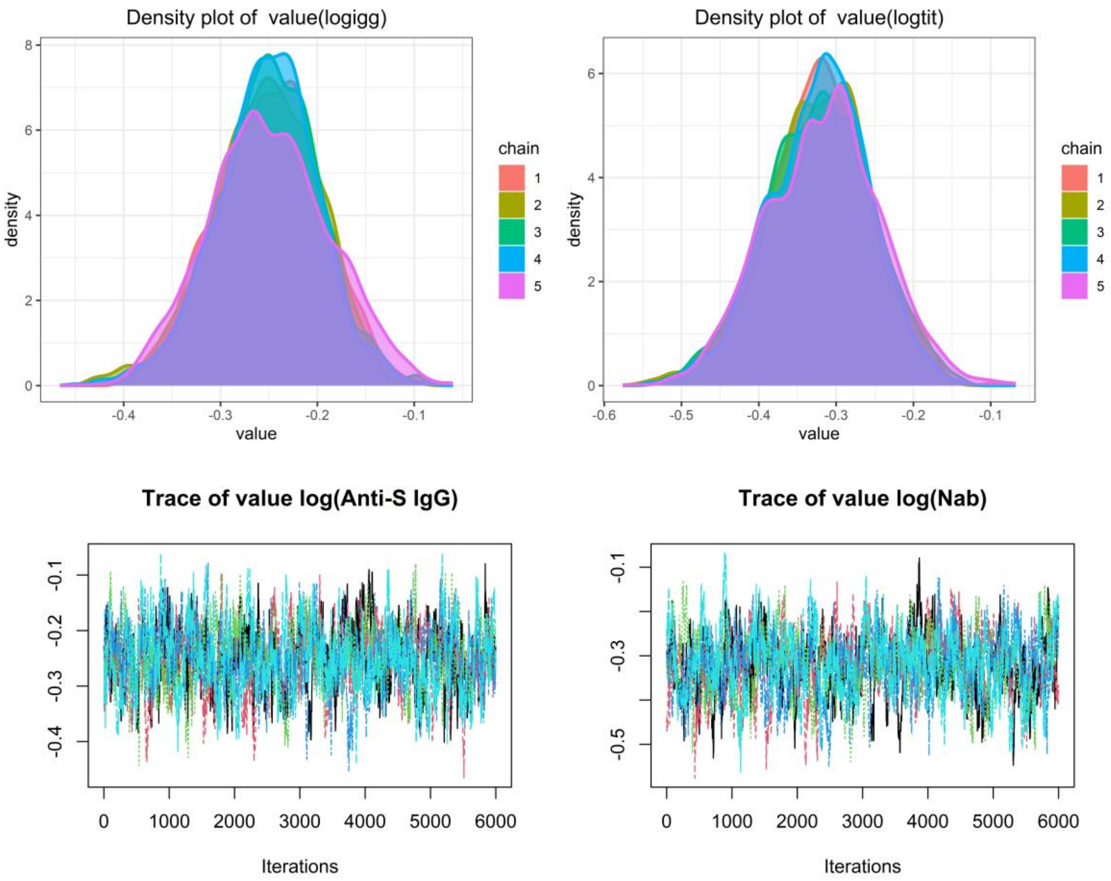


**Fig. S13:** Diagnostics for the two joint models

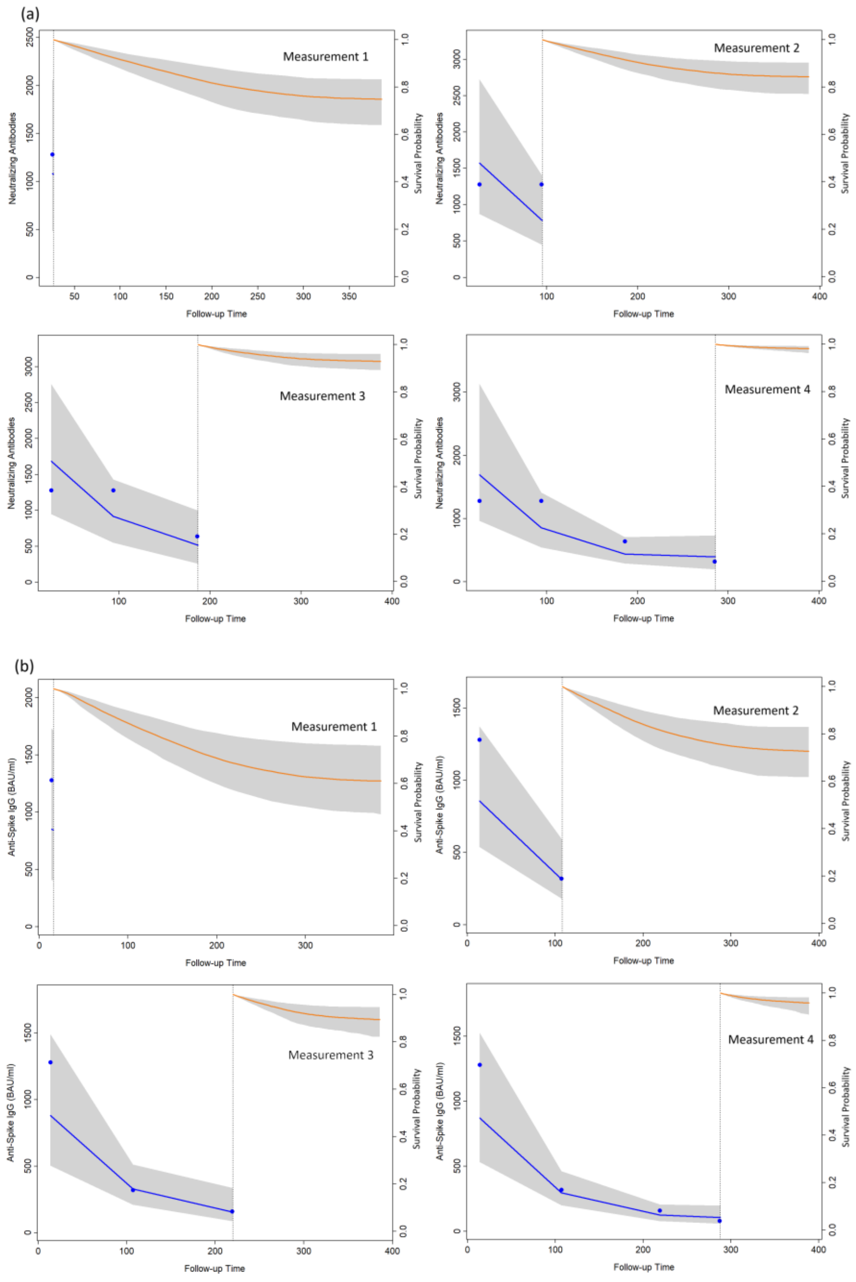


**Fig. S14: Two cases of dynamic longitudinal changes in the Neutralizing antibody titers and Anti-Spike IgG (left to the dashed line: point, measurement result; blue line, fitted trend) and the corresponding change in the predicted conditional overall survival probabilities (right to the dashed line: red line, conditional overall survival curve; gray area, 95% confidence interval) for two specific participants (a) and (b) respectively after the booster dose administration.** Participant **(a)** is a 59-year-old male who has received the primary vaccination with BNT162b2. He underwent a booster shot with BNT162b2 193 days after the initial vaccination and had not been previously infected before receiving the booster dose. Participant **(b)** is a 63-year-old male who has received the primary vaccination with ChAdOx1. He underwent a booster shot with BNT162b2 144 days after the initial vaccination and had not been previously infected before receiving the booster dose.

Supplementary material

| Table S1. Median Absolute errors (MAE) with Q1 and Q3 percentiles and Root Mean Square Error (RMSE) corresponding to Fig. 4(d) and 4(h). | | | | | | | | |
|---|---|---|---|---|---|---|---|---|
| Measurements | JM (Nab) | | LME (Nab) | | JM (IgG) | | LME (IgG) | |
| | MAE (Q1-Q3) | RMSE | MAE (Q1-Q3) | RMSE | MAE (Q1-Q3) | RMSE | MAE (Q1-Q3) | RMSE |
| 1 | 0.19 (0.10-0.29) | 0.27 | 0.20 (0.12-0.34) | 0.32 | 0.05 (0.03-0.09) | 0.09 | 0.08 (0.04-0.15) | 0.15 |
| 2 | 0.16 (0.08-0.27) | 0.24 | 0.22 (0.11-0.42) | 0.36 | 0.05 (0.03-0.09) | 0.08 | 0.12 (0.06-0.15) | 0.20 |
| 3 | 0.14 (0.07-0.24) | 0.20 | 0.19 (0.09-0.34) | 0.29 | 0.04 (0.02-0.07) | 0.07 | 0.06 (0.03-0.11) | 0.13 |
| 4 | 0.12 (0.06-0.18) | 0.16 | 0.18 (0.11-0.29) | 0.30 | 0.03 (0.01-0.05) | 0.04 | 0.08 (0.04-0.14) | 0.12 |
| 5 | 0.09 (0.04-0.12) | 0.11 | 0.37 (0.08-0.79) | 0.57 | 0.02 (0.01-0.04) | 0.03 | 0.05 (0.02-0.06) | 0.07 |

| Table S2. Prediction accuracy metrics at varying times. | | | | | | | | | |
|---|---|---|---|---|---|---|---|---|---|
| | | Nab – Joint model 1 | | | | Anti-S IgG – Joint model 2 | | | |
| Still at Risk | [t, u] | AUC | Cut-off | Sensitivity | Specificity | AUC | Cut-off | Sensitivity | Specificity |
| 672 | [30, 240] | 0.663 | 0.69 | 0.633 | 0.629 | 0.670 | 0.65 | 0.519 | 0.742 |
| 725 | [60, 240] | 0.651 | 0.73 | 0.619 | 0.621 | 0.659 | 0.74 | 0.747 | 0.512 |
| 691 | [90, 240] | 0.652 | 0.77 | 0.612 | 0.640 | 0.662 | 0.78 | 0.731 | 0.527 |
| 558 | [120, 240] | 0.689 | 0.82 | 0.727 | 0.581 | 0.689 | 0.80 | 0.625 | 0.666 |
| 511 | [150, 240] | 0.668 | 0.87 | 0.706 | 0.544 | 0.663 | 0.85 | 0.606 | 0.674 |
| 240 | [180, 240] | 0.673 | 0.91 | 0.628 | 0.650 | 0.678 | 0.89 | 0.514 | 0.787 |
| 342 | [210, 240] | 0.713 | 0.96 | 0.747 | 0.596 | 0.734 | 0.95 | 0.649 | 0.767 |
| 322 | [220, 260] | 0.760 | 0.95 | 0.835 | 0.626 | 0.767 | 0.94 | 0.674 | 0.758 |
| 672 | [30, 60] | 0.654 | 0.90 | 0.562 | 0.716 | 0.624 | 0.91 | 0.562 | 0.643 |
| 725 | [60, 90] | 0.572 | 0.94 | 0.899 | 0.221 | 0.587 | 0.92 | 0.728 | 0.439 |
| 691 | [90, 120] | 0.615 | 0.93 | 0.817 | 0.365 | 0.618 | 0.92 | 0.747 | 0.482 |
| 558 | [120, 165] | 0.685 | 0.92 | 0.754 | 0.555 | 0.676 | 0.92 | 0.755 | 0.511 |
| 511 | [150, 195] | 0.637 | 0.90 | 0.517 | 0.730 | 0.636 | 0.92 | 0.652 | 0.571 |
| 470 | [180, 225] | 0.647 | 0.93 | 0.640 | 0.590 | 0.661 | 0.93 | 0.658 | 0.580 |
| 672 | [30, 90] | 0.642 | 0.89 | 0.67 | 0.577 | 0.627 | 0.88 | 0.567 | 0.646 |
| 725 | [60, 120] | 0.590 | 0.90 | 0.687 | 0.461 | 0.592 | 0.89 | 0.653 | 0.509 |
| 691 | [90, 150] | 0.648 | 0.90 | 0.731 | 0.518 | 0.650 | 0.90 | 0.796 | 0.441 |
| 558 | [120, 180] | 0.676 | 0.89 | 0.671 | 0.616 | 0.670 | 0.90 | 0.781 | 0.490 |
| 511 | [150, 210] | 0.628 | 0.89 | 0.543 | 0.665 | 0.622 | 0.89 | 0.558 | 0.652 |
| 470 | [180, 240] | 0.673 | 0.91 | 0.628 | 0.650 | 0.678 | 0.89 | 0.514 | 0.787 |
| [t, u], u>t, u is the time of the obtained prediction from present time t; AUC is the time-dependent area under the receiver operating characteristic curve (ROC-curve); Sensitivity and Specificity are reported for the optimal threshold (Youden index); | | | | | | | | | |
